# Biodegradation of bovine spongiform encephalopathy prions in compost

**DOI:** 10.1038/s41598-022-26201-2

**Published:** 2022-12-23

**Authors:** Shanwei Xu, Sujeema Abeysekara, Sandor Dudas, Stefanie Czub, Antanas Staskevicius, Gordon Mitchell, Kingsley K. Amoako, Tim A. McAllister

**Affiliations:** 1 Agriculture and Agri-Food Canada, Morden Research and Development Centre, 101 Route 100, Morden, MB R6M 1Y5 Canada; 2 Agriculture and Agri-Food Canada, Lethbridge Research and Development Centre, Lethbridge, AB Canada; 3 Canadian and WOAH Reference Laboratories for BSE, Canadian Food Inspection Agency, Lethbridge, AB Canada; 4 Canadian and WOAH Reference Laboratories for Scrapie and CWD, Canadian Food Inspection Agency, Ottawa, ON Canada; 5 National Centres for Animal Disease, Canadian Food Inspection Agency, Lethbridge, AB Canada

**Keywords:** Pathogens, Environmental impact

## Abstract

To reduce the transmission risk of bovine spongiform encephalopathy prions (PrP^BSE^), specified risk materials (SRM) that can harbour PrP^BSE^ are prevented from entering the feed and food chains. As composting is one approach to disposing of SRM, we investigated the inactivation of PrP^BSE^ in lab-scale composters over 28 days and in bin composters over 106–120 days. Lab-scale composting was conducted using 45 kg of feedlot manure with and without chicken feathers. Based on protein misfolding cyclic amplification (PMCA), after 28 days of composting, PrP^BSE^ seeding activity was reduced by 3–4 log_10_ with feathers and 3 log_10_ without. Bin composters were constructed using ~ 2200 kg feedlot manure and repeated in 2017 and 2018. PMCA results showed that seeding activity of PrP^BSE^ was reduced by 1–2 log_10_ in the centre, but only by 1 log_10_ in the bottom of bin composters. Subsequent assessment by transgenic (Tgbov XV) mouse bioassay confirmed a similar reduction in PrP^BSE^ infectivity. Enrichment for proteolytic microorganisms through the addition of feathers to compost could enhance PrP^BSE^ degradation. In addition to temperature, other factors including varying concentrations of PrP^BSE^ and the nature of proteolytic microbial populations may be responsible for differential degradation of PrP^BSE^ during composting.

## Introduction

Transmissible spongiform encephalopathies (TSE) are fatal neurodegenerative diseases including scrapie in sheep and goats, chronic wasting disease (CWD) in cervid species, bovine spongiform encephalopathy (BSE) in cattle, and Creutzfeldt-Jackob disease (CJD) in humans. These diseases arise as a result of conformational changes in normal cellular prion proteins (PrP^C^) into misfolded-infectious prion proteins (PrP^TSE^)^1^. To date, 20 cases of BSE have been confirmed in Canada^2,3^ and in 2007, the Canadian Food Inspection Agency (CFIA) imposed an enhanced feed ban that excluded the specified risk materials (SRM; i.e., skull, brain, trigeminal ganglia, eyes, palatine tonsils, spinal cord and dorsal root ganglia of cattle 30 months or older, as well as the distal ileum from cattle of all ages) from the entire terrestrial and aquatic animal feed chains and fertilizers. Currently, approximately 250,000 tonnes of SRM are generated in Canada annually^4^ and its proper disposal is critical to the efforts of regulatory agencies to curtail BSE.

In Canada, the majority of SRM are rendered, dehydrated and disposed of in landfills^5,6^, a practice that does not necessarily inactivate all BSE prions (PrP^BSE^)^7^. Disposal of SRM in a manner that inactivates PrP^BSE^ is challenging due to the recalcitrant nature of this misfolded protein^8^. Current disposal practices approved by CFIA^9^ for PrP^TSE^ include incineration at 850°, 1-h disinfection with 2 N sodium hydroxide, autoclaving in saturated steam at 134°C for 60 min, thermal hydrolysis at 180°C under 12 atmpospheric pressure for no less than 40 min, and alkaline hydrolysis at 150°C and 4 atm with 15% sodium hydroxide or 19% potassium hydroxide (w w^−1^) for at least 180 min. All of these methods are costly due to the level of energy and complex infrastructure that they require to inactivate PrP^BSE^. Many of these processes also generate waste streams that require further treatment prior to release into the environment.

Composting is an aerobic process whereby organic matter including proteins are degraded by the actions of mesophilic and thermophilic bacteria and fungi. Previous research^10^ has shown that proteinases produced by *Streptomyces*, *Thermus*, *Bacillus*, and *Tritirachium* can degrade PrP^TSE^. Bacteria with high proteolytic activity have been isolated from compost^11,12^ and a vast array of proteases have been identified^13,14^. Compost is highly alkaline (pH 8–10) and temperatures can exceed 55ºC for weeks or months^15^, conditions that promote protein denaturation and hydrolysis. Long term exposure to proteases under these conditions raises the possibility that the microbial consortia in compost may have the capacity to degrade PrP^BSE^. Achieving thermophilic temperatures for a prolonged duration may be a key indictor of the potential to biodegrade SRM and inactivate PrP^BSE^ in compost^16,17^.

Previously, our research group used protein misfolding cyclic amplification (PMCA) to confirm a reduction of PrP^TSE^ seeding activity with 2 log_10_ in hamster 263K prions (PrP^263^^K^) and 3 log_10_ in CWD prions (PrP^CWD^) after 28 days in lab-scale composters^18^. In this same study, we also measured a 4.8 log_10_ reduction in PrP^263K^ infectivity based on hamster bioassays after 230 days of field-scale composting. In this study, we (1) assessed the biodegradation of PrP^BSE^ in lab-scale composters using PMCA and (2) in large bin composters in containment to estimate the degradation of PrP^BSE^ using PMCA and an infectivity bioassay. The compost bin model was subjected to multiple heating cycles to generate a thermophilic composting period that was similar to our previous field-scale studies^18^ which resulted in a 4.8 log_10_ reduction in PrP^263K^ infectivity.

## Materials and methods

### Ethics statement

In this study, animal bioassay work was conducted at the CFIA, Ottawa Laboratory-Fallowfield. Manure generated from pathogen free beef cattle at the Lethbridge Animal Disease Research Institute feedlot was collected and used for the bin composting experiment. The authors declare that the study is reported in accordance with ARRIVE guidelines. All animal associated work was performed in compliance with Canadian Council on Animal Care guidelines^19^. Protocols were assessed and approved by the Animal Care Committee at the CFIA, Ottawa Laboratory-Fallowfield (Animal Use Protocol ACC # 18-05).

### Sources of tissues and PrP^BSE^

Infectious bovine brain tissues used in this study were derived from two different sets of experimentally BSE-infected cattle, which were either orally or intracerebrally challenged with classical (i.e., C-type) BSE from a Canadian field case. Specifically, BSE infected brain tissues taken from a cattle after primary passage of this BSE field case through intra-cerebral challenge over 26 months were used in lab-scale composting experiment. Brain tissues used in the field-scale composting experiment were derived from a cattle orally challenged for approximately 50 months, using the brain materials from the intra-cerebral primary passaged cattle mentioned above. Positive tissues used in both lab-scale and bin composting experiments were confirmed with high level of C-type PrP^BSE^ in the brainstem by immunochemical and histopathological techniques at the Canadian and WOAH Reference Laboratories for BSE in Lethbridge, AB (Supplementary Fig. S1). The main reason for using different sources of BSE positive brains for two composting experiments was limitations in the volume of materials available. We were unsure if sufficient intra-cerebral challenged brain materials were available for the field-scale composting experiment so we elected to use brains from the primary passage of the intra-cerebrally challenged animal. The homogenate pools used for composting experiments required the use of brainstem as well as other brain areas. Brain tissues from non-infected BSE cattle, confirmed via immunohistochemistry, served as negative controls in studies. Tgbov XV mouse brain tissues, which are engineered to over-express bovine PrP^C^, were used for the PMCA analysis and obtained from CFIA, Ottawa Laboratory-Fallowfield (originally acquired from the Friedrich-Loeffler-Institute, Isle of Reims, Germany). All brain tissues were homogenized (1 g + 9 mL) in phosphate buffered saline (PBS) using the PrioGENIZER™ homogenizer (Prionics AG, Zurich, Switzerland) to yield a 10% brain homogenate (BH).

### Lab-scale composting experiment

Lab-scale compost experiments were conducted as described by Xu et al.^18^. Briefly, PrP^BSE^ was composted in passively aerated lab-scale composters filled with a mixture of cattle manure and wood shavings with or without poultry feathers obtained from a commercial abattoir. The fresh cattle manure was generated from cattle at the Agriculture and Agri-Food Canada feedlot in Lethbridge, AB. The compost mixture consisted of 72% (w w^−1^; dry basis) cattle manure and 28% wood shavings, while 5% poultry feathers were substituted for manure in the feather enriched treatment. The composting experiment was conducted in a level 3 containment laboratory at CFIA in Lethbridge, AB with duplicate composters per treatment. The PrP^BSE^ was introduced into compost by inoculating dried manure spheres (1.0 ± 0.1 g; dry basis) with 1 mL of a 10% BH solution containing PrP^BSE^. Subsequently, each inoculated manure sphere was sealed in a nylon bag (53 μm pore size; ANKOM Technology, Macedon, USA). Two replicate nylon bags were then placed in a larger polyester mesh bag (5 mm pore size) along with 200 g of the composting mixture. Polyester twine was attached to each mesh bag to enable recovery of samples from composters. As each composter was filled, duplicate PrP^BSE^ mesh bags were placed at a depth of 30 cm below the surface of the composting mixture. After 14 days, one mesh bag containing PrP^BSE^ was randomly collected. After collection, each composter was emptied, and contents were then mixed with water to return the compost to 60–70% moisture. Compost was then returned to its original composter for a second heating cycle. As the composters were refilled, the remaining PrP^BSE^ mesh bags were placed in each composter at the same depth as in the first cycle. After 28 days, the second mesh bag was collected. Fresh and composted PrP^BSE^ inoculated manure spheres were collected and stored at −80°C for PrP^BSE^ extraction and PMCA analysis.

### Bin model composting experiment

To assess inactivation of PrP^BSE^ in large bin composters, two bins were established at the CFIA Lethbridge biocontainment level 3 facility (Supplementary Fig. S2a) and the experiment was repeated in 2017 and 2018. As with the laboratory-scale composters, compost bins were mixed to promote multiple heating cycles, so as to generate a thermophilic period (≥55°C) similar to that achieved with PrP^263K^ in field-scale composters^18^. Compost bins (1.8 m length × 1.8 m width × 1.2 m height) were constructed using metal fence panels (Fig. S2a) surrounded with plastic snow fence so as to retain the compost matrix within each bin (Fig. S2b). During compost pile construction, ~10 cm of wood shavings were placed in the bottom of each bin to absorb leachate (Fig. S2b). Subsequently, ~ 2200 kg of mixed fresh manure from pathogen free cattle at the CFIA Lethbridge feedlot was placed on top of the wood shavings, to form a dome shaped compost pile with a height of 1.5 m (Fig. S2b).

During construction of compost piles, thermal couples connected to a data logger (CR850; Campbell Scientific, Edmonton, AB) were placed at nine locations (Location 1–9 across from edge to core part; Fig. S2c) at each of three depths (top, centre and bottom; total 27 locations) from the surface (36, 72, and 108 cm) to measure hourly temperature. When temperature declined to below 50°C in the centre of the bin composters (i.e., Location 5; Fig. S2c), each compost pile was mixed and transferred to a secondary bin to initiate the next heating cycle. For optimal composting, water, wood shavings and fresh manure were added to the compost piles at each mixing so as to maintain the moisture and C/N ratios within a range of 60–70% and 20–25, respectively. Triplicate samples of fresh manure used in each compost pile were collected for subsequent physicochemical analysis as described by Xu et al.^20^. A total of three heating cycles were generated over the experiment (Table 1).

**Table 1:** Total duration, mixing dates and initial manure properties for lab-scale and bin composting experiments.

Compost constituent	Lab-scale composters^a^		Bin composters	
	Control compost	Feather compost	2017 compost piles	2018 compost piles
Total duration days	28	28	106	120
Mixing dates	Day 14	Day 14	Days 44 and 78	Days 41 and 75
Duration of temperature ≥ 55°C^b^	2 days before mixing (centre)	2 days before mixing (centre)	67 days (centre)	80 days (centre)
	2 days after mixing (centre)	< 1 day after mixing (centre)	72 days (top)	74 days (top)
			31 days (bottom)	39 days (bottom)
Moisture (%)	60.7 ± 0.3b^c,d^	62.3 ± 0.5b	71.9 ± 0.2a	71.9 ± 1.5a
pH	8.3 ± 0.0c	8.2 ± 0.0d	8.7 ± 0.0a	8.5 ± 0.0b
Total carbon (%)	45.2 ± 0.2a	46.0 ± 0.2a	36.1 ± 1.0b	35.8 ± 1.5b
Total nitrogen (%)	2.2 ± 0.1b	2.8 ± 0.0a	2.1 ± 0.1b	2.0 ± 0.1b
C/N ratio	21.1 ± 1.0a	16.4 ± 0.2b	16.9 ± 0.9b	18.0 ± 0.2b

^a^Data was cited from Xu et al.^18^.

^b^Duration of temperature ≥55°C at the centre layer of lab-scale composters and at the core location (Location 5 in Fig. S2c) of top, centre and bottom layers of bin composters.

^c^Mean ± SEM (n = 4 for lab-scale compost; n = 6 for bin compost).

^d^Within a row, values followed by different lower case letters differ (*P* < 0.05) between lab-scale and bin composting experiments.

To assess the degradation of PrP^BSE^ using PMCA, two mesh bags with each bag containing one PrP^BSE^ inoculated manure sphere (1.0 ± 0.1 g, DM basis) were placed in the centre layer (i.e., Location 5 in Fig. S2c) of each bin composter in 2017 using the mesh bag method as per the lab-scale composters. In 2018, mesh bags containing manure spheres were placed at both centre and bottom layers, to assess the impact of location on PrP^BSE^ degradation. In addition, stainless steel suture wires were coated with PrP^BSE^ and placed within the bin composters to assess the impact of composting on PrP^BSE^ infectivity using a Tgbov XV mouse bioassay. Stainless steel wires were inoculated with PrP^BSE^ in a manner similar to Pritzkow et al.^21^. Briefly, stainless steel wires (3-0 monofilament; 4 mm; SMI AG, St. Vith, Belgium) were washed in 2% Triton X-100 for 15 min with sonication (Sonorex RK102 P; Bandelin Electronics, Berlin, Germany) at 102 Watts and then rinsed in distilled water, dried, and sterilized in a steam autoclave at 121°C for 20 min. In batches of 12, steel wires were incubated in 100 µL of 10% PrP^BSE^ BH for 2 h, with constant shaking at 500 rpm in a thermomixer, followed by storage at 4ºC overnight. Steel wires were then washed three times in PBS buffer, sealed in nylon bags and placed within the compost bins. A total of two mesh bags, each containing 12 PrP^BSE^ coated steel wires were placed within each bin composter at the same location as the bags containing manure spheres in both 2017 and 2018. Care was taken to ensure that the wires were evenly spread out in the bags prior to implantation. Mesh bags containing manure spheres and stainless steel wires inoculated with 10% BH from PrP^BSE^-negative cattle were included in bin composters as negative controls. During mixing events, manure spheres and stainless steel wires were removed and subsequently returned to their original location in the next heating cycle. All mesh bags were retrieved from compost bins once the targeted duration of thermophilic composting was achieved. After retrieval, manure spheres and steel wires were removed from mesh bags and stored at −80°C until used for PMCA and the mouse bioassay. Triplicate compost samples were collected from each bin composter at establishment, after each mixing event and at the end of the experiment for analyses of moisture, pH, total carbon (TC) and total nitrogen (TN) as described by Xu et al.^20^. Due to restrictions on the transfer of samples from the biocontainment facility, only samples collected prior to composting were analyzed for TC and TN using an automated CNS analyzer (NA2100, Carlo Erba Strumentazione, Milan, Italy).

### Migration of PrP^BSE^ in compost

To assess if reductions of PrP^BSE^ in manure spheres in bin composters was due to dissemination of prions into the surrounding compost, as opposed to microbial degradation, multiple compost matrix samples adjacent to each nylon bag containing manure spheres and stainless steel wires were collected at the end of each experiment, combined and stored at −80°C. Composite samples (n = 8; 1.0 ± 0.1 g; DM basis) from both years were then extracted for detection of PrP^BSE^ as described below.

### PrP^BSE^ extraction

Fresh and composted manure spheres inoculated with positive and negative PrP^BSE^ collected from both the laboratory and bin composters were extracted for PMCA using a modification of the procedure of Xu et al.^18^. Briefly, 4.5 mL of a 1% aqueous solution of sodium dodecyl sulphate (SDS; Sigma-Aldrich, Oakville, ON) was added to each sample (1.0 ± 0.1 g; DM basis) in a 50 mL falcon tube and vigorously shaken on a platform shaker (Model 100; VWR, Mississauga, ON) for 2 h. To enhance the efficiency of PrP^BSE^ extraction, the mixture was transferred to a 30 mL syringe attached to a nylon filter (0.45 µm pore size; VWR, Mississauga, ON) to separate liquid from compost residues. The collected liquid was placed in a 50 mL falcon tube and centrifuged at 3,200 g for 20 min. Subsequently, 1.5 mL of supernatant was precipitated with sodium phosphotungstic acid (4%, w v^−1^, in 170 mM MgCl_2_, pH 7.4) to achieve a final concentration of 0.3% (w v^−1^) and centrifuged at 17,900*g* for 30 min. The precipitated protein pellet was re-suspended in 50 µL of 1× conversion buffer (PBS supplemented with 150 mM NaCl and 1% Triton X-100 with EDTA-free protease inhibitor) and stored at −80°C for subsequent analysis by PMCA.

Changes in chemical and physical properties of compost during the composting period may have influenced the extraction efficiency of PrP^BSE^. To test this possibility, fresh manure spheres without PrP^BSE^ were placed at the centre of each bin composter and collected after composting. These composted samples along with fresh manure spheres without PrP^BSE^ were inoculated with a log dilutions series (i.e., 10^–1^ to 10^–7^) of 10% PrP^BSE^ BH and extracted as described above, and recovery of PrP^BSE^ was assessed by PMCA.

### PMCA assay

For PMCA, a 10% non-infectious Tgbov XV BH solution containing a complete protease inhibitor cocktail (Roche Diagnostics, Laval, QC) was prepared as described by Ding et al.^22^. To verify PrP^BSE^ amplification using PMCA, a 10% BSE BH was serially diluted tenfold in 10% BSE-negative BH. Subsequently, 8 µL of each tenfold dilution and a negative control (10% BSE-negative BH) were mixed with 72 µL of 10% non-infectious Tgbov XV BH and then transferred to 0.2 mL PCR tubes and placed in a Misonix model Q700 sonicator (Misonix Inc., Farmingdale, NY). Samples were incubated in the sonicator horn with 200 mL of distilled water at 37°C and subjected to 144 cycles of 20-s sonication (140–185 W potency) followed by 29-min and 40-s of incubation. Two to three rounds of PMCA were performed under these conditions with each subsequent round using a 1:10 dilution of prion proteins from the previous amplified round as a template. To measure inactivation of PrP^BSE^ during composting, extracted fresh manure and compost samples were serially diluted tenfold with 10% non-infectious Tgbov XV BH and subjected to PMCA amplification using the same protocols as mentioned above. Due to the use of different sources of PrP^BSE^ in laboratory and bin composters, samples from the lab-scale composting experiment were subjected to two rounds of PMCA analysis, while three rounds of PMCA were used to analyze the bin compost samples. All PMCA products along with 10% non-infectious and infectious BH samples were then digested by proteinase K (117 µg mL^−1^) for 1 h at 48°C and PrP^BSE^ was detected using Western blotting (WB) as described by Xu et al.^23^, using mAb 6H4 (1:5000 dilution; Ovine PrP148-157; Prionics, Zurich, Switzerland). To test for false positive signals as described by Moudjou et al.^24^ and Cosseddu et al.^25^, duplicate BSE-negative samples of 10% Tgbov XV BH and extract from fresh manure spheres inoculated with PBS buffer and BSE negative bovine BH were subjected to five-rounds of PMCA.

### Bioassay

Tgbov XV mice (Friedrich-Loeffler-Institute, Isle of Reims, Germany) were used for the bioassay, as they overexpress bovine PrP^C^ and are highly susceptible to BSE infection^26^. To accurately calculate log_10_ reductions of PrP^BSE^ infectivity by composting, it was necessary to establish the relationship between the dose and time to disease in the Tgbov XV steel wire implantation model. Therefore, stainless steel wires coated with a tenfold serially diluted (10^–1^–10^–9^) PrP^BSE^ BH in PBS and 10% non-infectious BH were prepared in a similar manner as described by Pritzkow et al.^21^. Individual wires that had contact with 10^–1^ to 10^–9^ PrP^BSE^ BH or a BSE negative BH control were intracranially implanted into Tgbov mice, with up to eight individuals per dilution. Inoculated mice were monitored daily and were euthanized when they displayed clinical signs of TSE or were asymptomatic after 600 days post inoculation (dpi). The number of days between inoculation and euthanasia or death was considered the survival period used in data analysis. Disease transmission was confirmed by immunohistochemical staining of brain sections using an automated immunostainer (Ventana Medical Systems, Tucson, USA) and the monoclonal antibody SAF84 (Cayman Chemical, Ann Arbor, USA).

To determine if composting reduced PrP^BSE^ infectivity, groups of four to eight mice were intracranially inoculated with washed non-composted and composted stainless steel wires collected from the compost experiment in each year. Negative control wires which were composted but not coated with PrP^BSE^ were also included in the bioassays. Mice were euthanized and assessed for PrP^BSE^ by immunohistochemistry and assessed for pathology after displaying clinical disease or after 600 days post inoculation as described above.

### Statistical analysis

Changes in manure moisture content and pH during bin composting in 2017 and 2018 were analyzed using the Mixed procedure of SAS (Version 9.2; SAS Institute Inc., Cary, USA) with sampling time treated as a repeated measure, bin composter (n = 2) as an experimental unit and mesh bag (n = 2) as a replicate in the model. An analysis of variance for the effect of types of fresh manure used to construct lab-scale (n = 4) and bin (n = 6) composting experiments on moisture, pH, and TC and TN content was performed using SAS Mixed procedures. Main effects in the analyses mentioned above were considered to be statistically significant at *P* < 0.05.

The log_10_ reductions of PrP^BSE^ infectivity by bin composting were calculated from the clinical bioassay dpi data based on a standard curve of four-parameter logistic model, i.e., clinical dpi = d + (a − d)/(1 + (log_10_dilution/c)^b^) using the non-linear regression calculation in MyAssays software (MyAssays Ltd; East Sussex, UK). The effect of year on log_10_ reductions of PrP^BSE^ infectivity in the centre of the bin composters (n = 2) and the effect of sampling location on the log_10_ reduction of PrP^BSE^ infectivity in 2018 bin composters (n = 2) were tested using SAS Mixed procedures respectively with differences declared at *P* < 0.05.

## Results

### Lab-scale compost properties

**Figure 1:**
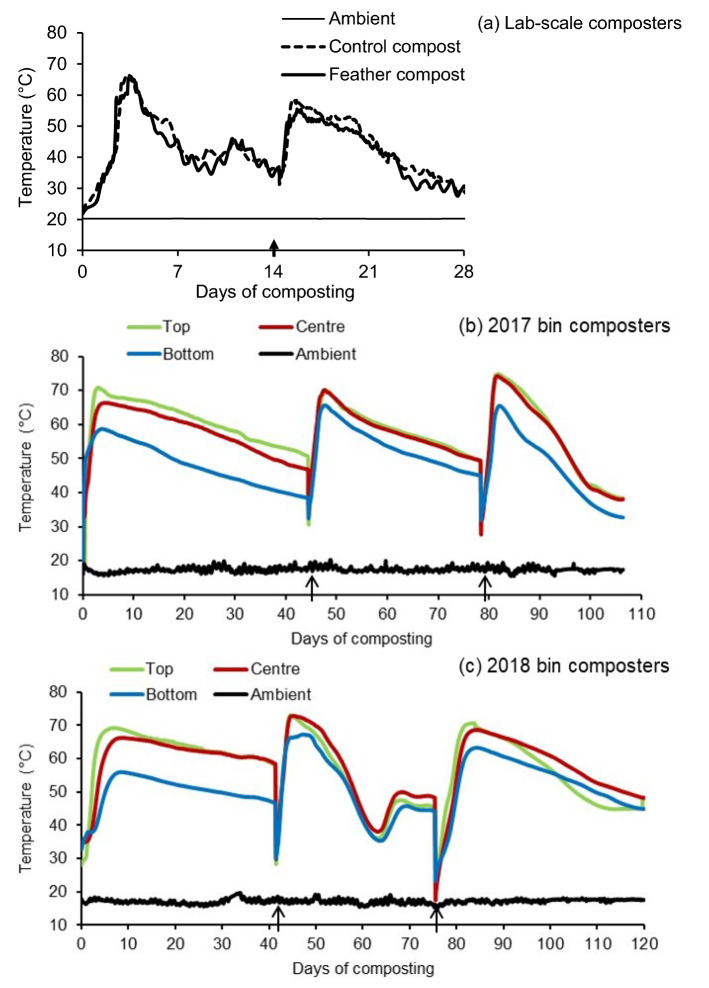
Temperature profiles from centre layer (40 cm height) of lab-scale composters (**a**) over 28 days and from the core location (i.e., Location 5 in Fig. S2) at top (108 cm), centre (72 cm) and bottom (36 cm) layers in bin composters over 106 days in 2017 (**b**) and 120 days in 2018 (**c**). Compost was mixed one time on day 14 for lab-scale composters and on days 44 and 78 in 2017 and days 41 and 75 in 2018 for bin composters. Arrows indicate the date compost was mixed. Lab-scale compost temperatures were originally reported in Xu et al.^18^.

Temperature profiles did not differ between control and feather compost in lab-scale composters (Fig. 1a). Both composts heated rapidly, peaking at 68°C in control compost and 66°C in feather compost. Temperatures remained above 55°C for 2 days in both composts during the first 14 days of composting (Table 1). After mixing and the addition of water, temperatures peaked at 59°C in the control compost and at 56°C in feather compost, while during the final 14 days of composting it remained above 55°C for 2 days in control compost and < 1 day in feather compost (Table 1).

### Bin compost properties

Temperature profiles in the core location (Location 5 in Fig. S2c) from the top, centre and bottom layers of bin composters were shown in Fig. 1b,c. Mixing and transferring procedures generated three heating cycles in both 2017 and 2018 bin composting experiments (Fig. 1b,c). In 2017, peak temperatures of 72°C, 70°C and 6 °C at the top, centre and bottom layers respectively, were achieved over three heating cycles (Fig. 1b). Temperatures remained ≥55°C for 72 days at the top layer, 67 days at the centre and 31 days at the bottom layer over 106 days in 2017 (Table 1). In 2018, over 120 days of composting, total duration of temperature ≥55°C at the top, centre and bottom layers were 74, 80 and 39 days, respectively (Table 1). Temperatures at the extremities (i.e., Locations 1–4 and 6–9 in Fig. S2c) of the bin composters in both of years varied by location, but lower peak temperatures and shorter duration of the temperature ≥55°C were observed compared to the core location of the piles at all three layers (Supplementary Figs. S3 and S4).

Feedlot manure used for bin composting in 2017 and 2018 had similar moisture content (71.9%), TC (36.0%) and TN (2.2%) content, and C/N ratio (17.4) except pH (8.5–8.7), but it remained higher (*P* < 0.0001) moisture and pH but lower (*P* < 0.001) TC content than that used in lab-scale composting experiment (Table 1). Changes in moisture content and pH of compost in bins were comparable in 2017 and 2018 (Supplementary Table S1). Moisture content gradually declined (*P* < 0.0001) after each mixing event with 52.4–54.5% at the end of the composting. Compost pH increased to 9.1–9.2 in both years after the first mixing and declined thereafter (Table S1). After the second mixing, compost in 2018 had a higher (*P* = 0.03) pH than 2017, but terminal compost pH was similar in both years, ranging from 8.4 to 8.6 (Table S1).

### Sensitivity of PMCA for measuring PrP^BSE^ in manure

To determine the PMCA detection limit of PrP^BSE^, fresh manure spheres inoculated with tenfold dilutions (i.e., 10^–1^ to 10^–6^) of 10% BSE BH used in lab-scale composting experiment were extracted and subject to PMCA analysis. PrP^BSE^ was detectable in a 10% infectious BH at dilution up to 10^–7^ after two rounds of PMCA (Supplementary Fig. S5). PrP^BSE^ in manure spheres was not detected before (Fig. S5a) or after (Fig. S5b) a single round of PMCA, but was detected at 3–4 log_10_ after two rounds of PMCA (Fig. S5c).

### Reduction of PrP^BSE^ in lab-scale composters measured by PMCA

**Figure 2:**
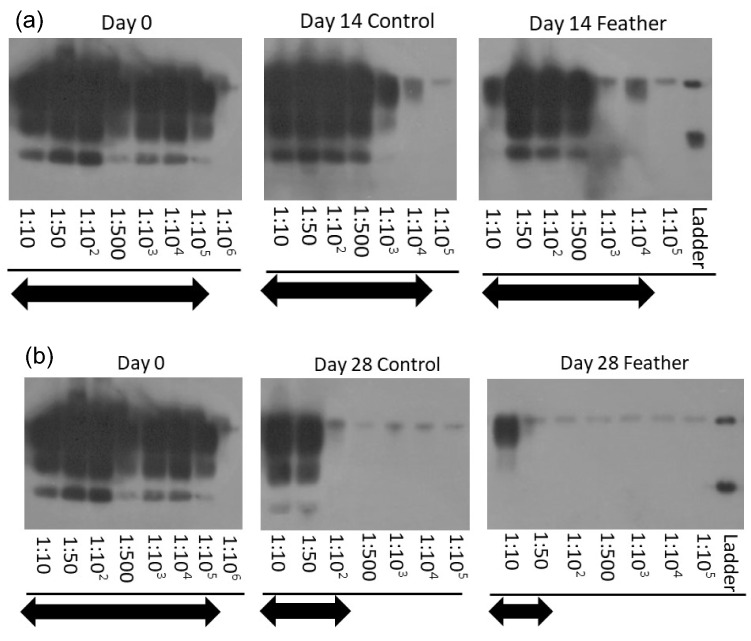
Representative Western blots of PrP^BSE^ extracted from manure spheres collected at days 0 and 14 (**a**) and at days 0 and 28 (**b**) from lab-scale composters after two rounds of protein misfolding cyclic amplification (PMCA). Control: control compost; Feather: feather compost. Arrows indicate the dilution range where PrP^BSE^ signals were detected. Molecular weight markers at 30 kDa (Upper ladder) and 20 kDa (Lower ladder) are indicated. The grouped blots for (**a**) were cropped from different parts of the same gel, while blots for (**b**) were cropped from different gels in parallel from the same PMCA and Western blot experiments. Original blots are presented in Supplementary Fig. S9.

Measurable signals of PrP^BSE^ were detected in fresh manure at dilutions up to 10^–5^ (Fig. 2). The seeding activity of PrP^BSE^ declined by 1 log_10_ in both control and feather compost after 14 days (Fig. 2a), and by 3 log_10_ in control compost and 3–4 log_10_ in feather compost after 28 days (Fig. 2b).

### Reduction of PrP^BSE^ in bin composters measured by PMCA

**Figure 3:**
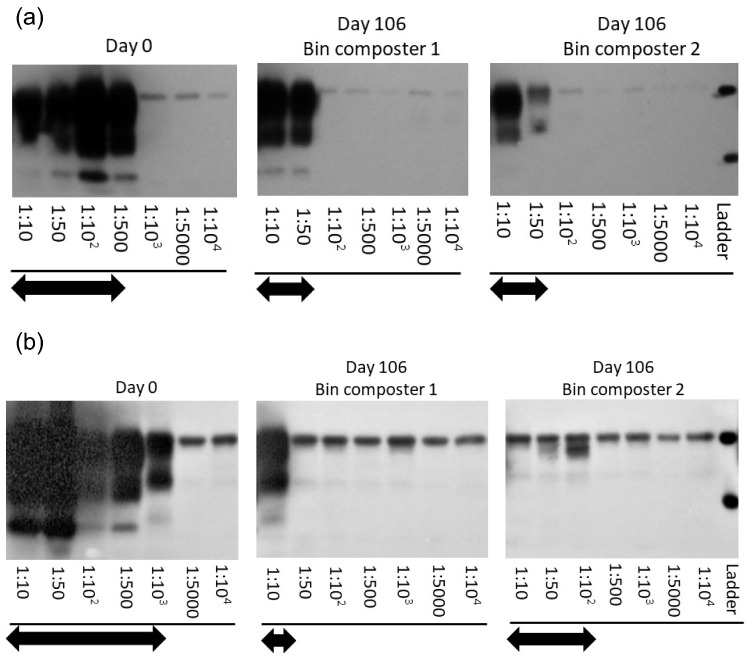
Representative Western blots of PrP^BSE^ extracted from duplicate manure spheres (**a,b**) collected at days 0 and 106 from the centre layer in 2017 bin composter 1 and 2 after three rounds of protein misfolding cyclic amplification (PMCA). Arrows indicate the dilution range where PrP^BSE^ signals were detected. Molecular weight markers at 30 kDa (upper ladder) and 20 kDa (lower ladder) are indicated. The grouped blots for each figure were cropped from different parts of the same gel. Original blots are presented in Supplementary Fig. S10.

**Figure 4:**
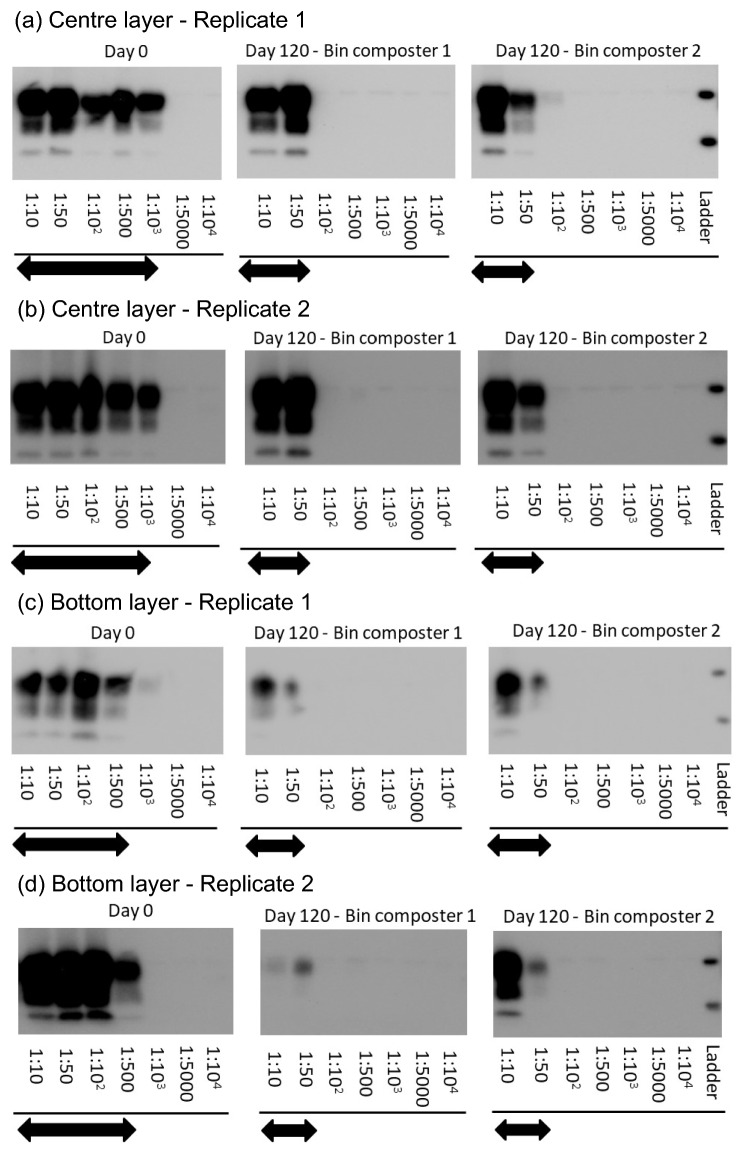
Representative Western blots of PrP^BSE^ extracted from duplicate manure spheres collected at days 0 and 120 from the centre (**a,b**) and bottom (**c,d**) layers in 2018 bin composters after three rounds of protein misfolding cyclic amplification (PMCA). Arrows indicate the dilution range where PrP^BSE^ signals were detected. Molecular weight markers at 30 kDa (upper ladder) and 20 kDa (lower ladder) are indicated. The grouped blots for each figure were cropped from different parts of the same gel. Original blots are presented in Supplementary Fig. S11.

PrP^BSE^ replication capacity was reduced by 1 log_10_ after 106 days at the centre layer of bin composters in 2017 (Fig. 3a). When the second replicate manure spheres were examined, seeding activity of PrP^BSE^ was reduced by approximately 1 log_10_ in Bin Composter 2, but by 2 log_10_ in Bin Composter 1 (Fig. 3b). In 2018, a similar 1–2 log_10_ reduction in PrP^BSE^ seeding capacity was detected at the centre layer after 120 days with the similar results between duplicate manure spheres (Fig. 4a,b). However, both bin composters had 1 log_10_ reduction of PrP^BSE^ seeding activity at the bottom layer after 120 days of composting (Fig. 4c,d). No PrP^BSE^ signals were detected after three rounds of PMCA in any of the eight compost samples that were collected from the immediate exterior of the nylon bags containing either PrP^BSE^ manure spheres or steel wires (Supplementary Fig. S6).

In the bin composting study, PrP^BSE^ BH was inoculated into fresh and composted manure spheres to test if dynamic changes in manure properties over the composting period influenced the PrP^BSE^ extraction efficiency. The results showed that fresh and composted samples differed in their limit of detection at 3 log_10_ and 5 log_10_ after three rounds of PMCA, respectively (Supplementary Fig. S7b). For the detection of de novo PrP^BSE^ during PMCA analysis, false positive signals only occurred in Tgbov BH and BSE negative BH extract samples after five rounds of PMCA (Supplementary Fig. S8).

### Inactivation of PrP^BSE^ in bin composters as measured by bioassay

For the titration assay conducted in 2017, all mice implanted with PrP^BSE^ stainless steel wires developed disease, with an increase in the duration to clinical disease from 425 ± 14 to 462 ± 15 dpi with increasing dilution (Supplementary Table S2). At a dilution of 10^–6^, 88% of the mice succumbed to disease, exhibiting clinical signs after 544 ± 26 dpi. No mice developed clinical symptoms of TSE from dilutions of 10^–7^ to 10^–9^ after 600 dpi (Table S2). In 2018, more than 75% of the mice developed disease at dilutions of 10^–1^ to 10^–3^ with an increase dpi from 385 ± 8 to 539 ± 25 (Table S2). In the remaining dilution groups, only one mouse at the 10^–5^ dilution tested positive for TSE at 525 dpi.

To determine the relationship between disease onset and log dilution, four-parameter logistic regression models were fitted (R^2^^2^ = 0.96 ~ 0.99) to data from the titration assay and used in subsequent calculations. Composting for 106 days resulted in a 0.4–1.3 log_10_ reduction in the infectivity of PrP^BSE^ arising from steel wires implanted at the centre layer of bin composters in 2017 (Table 2). A similar reduction (0.6–1.1 log_10_) in infectivity was observed for PrP^BSE^ wires that were implanted at this location and composted for 120 days in 2018 (Table 3). However, compost collected from the bottom layer resulted in less (*P* = 0.04) of a reduction (0.3–0.7 log_10_) in PrP^BSE^ infectivity than the centre layer in 2018 (Table 3).

**Table 2:** Inactivation of PrP^BSE^ bound to stainless steel wires in 2017 bin composters.

Treatment	Tgbov XV mice		
	Survival period in dpi^a^ (mean ± SEM)	Attack rate^b^	Infectivity reduction (log_10_)
**Bin composter 1**			
Positive wires—Day 0	439 ± 17	4/4	
Positive wires—Day 106	458 ± 17	7/7	1.3
Negative wires—Day 0		0/3	
Negative wires—Day 106		0/5	
**Bin composter 2**			
Positive wires—Day 0	459 ± 6	4/4	
Positive wires—Day 106	473 ± 13	8/8	0.4
Negative wires—Day 0		0/4	
Negative wires—Day 106		0/6	

^a^Survival period: number of days post inoculation (dpi) when mice died or were euthanized with clinical signs of neurological disease. Mice euthanized due to concurrent illness or that tested TSE-negative were not included in calculations. Mean values are not presented if all mice in a group were TSE-negative.

^b^Attack rate: number of TSE-positive mice / Number of mice challenged.

**Table 3:** Inactivation of PrP^BSE^ bound to stainless steel wires in 2018 bin composters.

Treatment and location	Tgbov XV mice		
	Survival period in dpi^a^ (mean ± SEM)	Attack rate^b^	Infectivity reduction (log_10_)^c^
Positive wires—Day 0	405 ± 17	12/12	
Negative wires—Day 0		0/9	
**Bin composter 1 (bottom)**			
Positive 1—Day 120	495 ± 10	8/8	0.7
Positive 2—Day 120	445 ± 16	8/8	0.4
Negative 1—Day 120		0/5	
Negative 2—Day 120		0/7	
**Bin composter 2 (bottom)**			
Positive 1—Day 120	459 ± 11	7/7	0.5
Positive 2—Day 120	429 ± 20	6/6	0.3
Negative 1—Day 120		0/5	
Negative 2—Day 120		0/6	
**Averaged infectivity reduction (log**_**10**_**) (bottom)**			0.5b
**Bin composter 1 (centre)**			
Positive 1—Day 120	494 ± 12	7/7	0.7
Positive 2—Day 120	532 ± 17	6/7	1.1
Negative 1—Day 120		0/5	
Negative 2—Day 120		0/5	
**Bin composter 2 (centre)**			
Positive 1—Day 120	500 ± 14	5/7	0.7
Positive 2—Day 120	488 ± 8	7/8	0.6
Negative 1—Day 120		0/7	
Negative 2—Day 120		0/5	
**Averaged infectivity reduction (log**_**10**_**) (centre)**			0.8a

^a^Survival period: number of days post inoculation (dpi) when mice died or were euthanized with clinical signs of neurological disease. Mice euthanized due to concurrent illness or that tested TSE-negative were not included in calculations. Mean values are not presented if all mice in a group were TSE-negative.

^b^Attack rate: number of TSE-positive mice/number of mice challenged.

^c^Within the column of infectivity reduction, values followed by different lower case letters differ (*P* < 0.05) between sampling locations within bin composters.

## Discussion

Improving on analytical sensitivity, amplification assays such as PMCA have been used to detect trace amounts of PrP^TSE^ in saliva, tongue, nasal mucosa, palatine tonsils, lymph nodes, ileocecal and muscular tissues of TSE-infected animals^27–29^. However, quantification of PrP^TSE^ in compost presents a significant challenge, due to the highly hydrophobic nature of PrP^TSE^ and their tendency to aggregate into plaque-like complexes that exhibit a high affinity for soil particles^30–32^. Among detergents, SDS has been used to release PrP^TSE^ from soil, manure, wood, metal and plastic for subsequent detection by WB^18,33,34^. Therefore, incorporation of PMCA after SDS extraction of PrP^TSE^ could provide an ultrasensitive method to quantify PrP^TSE^ degradation in compost. Currently, PMCA has been successfully applied to examine the contamination and persistence of PrP^TSE^ in soil, wheat grass, wood, plastic, cement, metals, stream and wastewater^35–38^. Our research team successfully employed PMCA with SDS extraction to quantify a declined PrP^TSE^ seeding activity with 2 log_10_ reduction in PrP^263^^K^ and 3 log_10_ reduction in PrP^CWD^ after 28 days of lab-scale composting^18^. However, we are unaware of studies that have used PMCA to assess the potential degradation of PrP^BSE^ in compost. In the current study, we established a PMCA assay that could detect manure-bound PrP^BSE^ at a 3–4 log_10_ sensitivity after two rounds of PMCA, enabling us to estimate the biodegradation of PrP^BSE^ in both lab and bin composters.

Animal bioassays represent the most definitive test for assessing the inactivation of PrP^TSE^. Although bioassays are expensive and require a considerable period of time to generate results^39^, their ability to measure the infectivity of prions is required to comprehensively assess PrP^TSE^ infectivity after composting and to substantiate PMCA results. Stainless steel has a high affinity for prions, which can result in the transmission of prion disease from contaminated surgical instruments^40,41^. Our titration results indicated that the bioassay remained sensitive to wire-bound dilutions up to 10^–3^ to 10^–6^, with an average clinical dpi of ~ 400 to 500 days, a detection limit similar to our PMCA protocol. Moreover, our bioassay showed a 1–2 log_10_ reduction of PrP^BSE^ infectivity in bin composters with a significantly higher reduction in the centre than the bottom layer, a finding that agrees with our PMCA observations. Similarly, Yoshioka et al.^42^ reported a similar level of PrP^BSE^ inactivation after heat treatment (140–180°C for 1–3 h) of yellow grease using both PMCA and Tgbov mouse bioassay. This suggests that PMCA may be reflective of the results obtained through bioassays, making an attractive alternative owing to its highly sensitive and comparatively rapid ability to detect PrP^BSE^.

Poultry feathers are predominantly composed of β-keratin (90% of DM), which similar to PrP^TSE^ are structurally rich in β-sheets^43,44^. Keratinases^10,12^ have the capacity to degrade feather protein and have shown activity against PrP^TSE^. Therefore, enrichment for keratinolytic microorganisms in compost through inclusion of feathers may be a means of promoting PrP^TSE^ degradation. In an earlier study by our group^18^, mixing feathers with cattle manure in lab-scale composters enhanced PrP^263^^K^ degradation by 1 log_10_ after 14 days and PrP^CWD^ by 0.5–1 log_10_ after 28 days. In the present study, there was also evidence that the degradation of PrP^BSE^ in lab-scale composters was enhanced by ~ 0.5 log_10_ as indicated from the different PrP^BSE^ seeding activity between compost extract samples with and without feathers after 28 days. Responses to the inclusion of feathers were more evident for PrP^CWD^ and PrP^BSE^ after the second composting cycle, suggesting that feathers may have selected for enriched prion degrading bacterial communities after a sufficient thermophilic period. Previous studies found that enrichment of a compost straw matrix with feathers not only effectively increased proteolytic activity in the early composting process but also promoted the growth of keratinolytic fungi during the latter stages of composting^45,46^. Currently, approximately 100,000 tonnes of feathers are produced from the Canadian poultry industry annually with most of them being landfilled or incinerated^47^. Feather composts have been recently considered to be used as agricultural fertilizers to promote crop production^48,49^ and share properties in common with slow release N fertilizers. Consequently, composting of SRM and feathers together might be an option for co-disposal of both agricultural waste materials generated from agriculture production with the potential co-benefit of maximizing PrP^BSE^ degradation in compost.

Composting of SRM has economic advantages as disposal is estimated at $120–180 CAN per tonne, two to three times lower than disposal methods such as landfill, incineration, or alkaline hydrolysis^50^. Composting also could be more amendable to remote areas where transport of SRM to a centralized processing facility is infeasible. Currently, CFIA regulations require at least 5 log_10_ inactivation of PrP^BSE^ to approve a method for SRM disposal. Our previous work demonstrated a 4.8 log_10_ reduction in PrP^263^^K^ infectivity after 230 days of field-scale static composting^18^. Turning of compost piles breaks up aggregates, increases porosity, redistributes moisture and promotes the microbial decomposition of organic matter, increasing the duration and temperature achieved during composting^16,18^. In practice, thermophilic composting (≥55°C) is recommended to inactivate environmental pathogens^51^ and guidelines from CCME (Canadian Council of Ministers of the Environment) and USEPA (United States Environmental Protection Agency) suggest temperatures should exceed 55°C for at least 15 consecutive days in windrows that are turned three times. Consequently, we elected to mix the compost to generate three heating cycles to subject PrP^BSE^ to a period of thermophilic exposure that was the same as what was achieved for PrP^263K^ in our static field-scale composters. In 2017 and 2018, similar sources of feedlot manure resulted in comparable changes in temperature, moisture and pH during the composting process. Both PMCA and bioassay results suggest that PrP^BSE^ was reduced by 1–2 log_10_ in the centre of compost piles in both years. In practice, compost piles consist of a heterogenous mixture of animal tissues, manure and a carbon source such as woodchips or straw. Even after blending, spatial variability in temperature profiles^15^ and the composition of microbial communities^52^ are often observed. In the present study, temperatures broadly varied among core and edge locations at all three depths of bin composters. In 2017, we did observe a large variation (1 log_10_ and 2 log_10_) in the reduction of PrP^BSE^ seeding activity between duplicate manure spheres at the centre of the same compost pile as measured by PMCA as well as in the reduction of PrP^BSE^ infectivity between bin composters (i.e., 0.4 log_10_ and 1.3 log_10_) as assessed by the bioassay. Therefore, the variability in matrix composition, microbial populations and temperature profiles generated in compost piles would make it virtually impossible to verify uniform degradation of PrP^BSE^ throughout a field scale compost pile. In addition, we did observe higher temperatures in the upper layers of bin composters as compared to the bottom. This temperature distribution likely reflects the upward movement of warm gases arising from microbial activity. Similar results were observed in our previous lab-scale composting study for degradation of SRM in compost^20^. As a result of this temperature variability, we examined the degradation of PrP^BSE^ at the centre and bottom compost layers in 2018. Both the PMCA and bioassay confirmed that the degradation of PrP^BSE^ was less at the bottom layer as compared to the centre layer after 120 days. This finding likely reflects the lower microbial activity at the bottom of compost piles as temperatures ≥55°C occurred for only 39 days at this location as compared to 80 days at the central location.

Several factors could significantly affect the accurate quantification of PrP^BSE^ biodegradation in compost. First, it is possible that PrP^BSE^ may have migrated from the manure spheres into the surrounding compost matrix or may be cleaved off the stainless steel wires without being degraded. We attempted to examine this possibility using PMCA to test for the presence of PrP^BSE^ in the compost matrix surrounding the nylon bags, but no signals were detected. Previous studies have shown that the mobility of prions is limited in environmental samples such as soil^7,53^, composted yard waste and municipal solid waste^54^. The mobility of PrP^TSE^ in these environmental samples was not observed even after exposure to stringent chaotropic agents, nonionic detergents or extreme pH^55,56^. These observations suggest that during composting, the mobility of PrP^BSE^ from manure spheres or their cleavage from stainless steel wires was unlikely. Moreover, Tgbov mice developed TSE disease prior to and after the composting process, suggesting PrP^BSE^ remained bound and infectious throughout composting. Thus, the reduction in PrP^BSE^ likely reflects enzymatic degradation.

With multiple cycles of PMCA, it is possible for false positives to be generated from non-infectious BH^24,25^. To check for false-positives, BSE-negative samples including Tgbov BH and extract samples from fresh manure spheres inoculated with non-infectious BH were subjected to multiple rounds of PMCA. False positive signals were only observed after the fifth round of PMCA. Consequently, we restricted PMCA to three rounds to ensure that declines in PrP^BSE^ seeding capacity during composting were not confounded by the spontaneous generation of false positive PrP^BSE^ signals. It is also possible that dynamic changes in manure properties during composting may have impacted the binding of PrP^BSE^ with manure/compost particles that could impact the subsequent PrP^BSE^ extraction or templating activity for PMCA detection. Ionic concentration and pH have been reported to influence PrP^TSE^ adsorption and conformational changes in soil^57,58^. Moreover, humic acid-prion interactions have also been shown to impact the extraction and detection of PrP^CWD^ in soils^59,60^. The disease-associated form of prions binding to soil particles or minerals were reported to either increase^61^ or decrease^62^ the prion infectivity and PMCA replication efficiency. Our results showed that compost-bound PrP^BSE^ enhanced PMCA replication efficiency than manure-bound PrP^BSE^ (i.e., 5 log_10_ vs 3 log_10_ limit of detection). This suggests that our established extraction protocols recovered more PrP^BSE^ from compost than fresh manure, resulting in more PrP^BSE^ seeds available for amplification. Another possible explanation could be made that changes in composition and structure of organic compounds such as humic acids during composting^63^ may modify the affinity of PrP^BSE^ to aged manure and enhance the ability of compost-bound prions to covert PrP^C^ to PrP^BSE^, resulting in a better propensity of compost-bound PrP^BSE^ to be amplified in our assay. However, these findings further support that 1–2 log_10_ reduction of PrP^BSE^ in bin compost is not due to differential PrP^BSE^ recovery or templating activity between manure and compost, but as a result of microbial activity during composting.

In the present study, lab-scale composters achieved ≥55°C for 3 days after two cycles over 28 days. This contrasts with bin composters where ≥55°C was achieved for 60–80 days after composting for 106–120 days. Our previous work demonstrated that PrP^263^^K^ was degraded during lab-scale composting and that degradation was enhanced as result of prolonged exposure to microbial activity in field-scale composters^18^. However, these differences in microbial activity in the current two composting systems did not result in more extensive inactivation of PrP^BSE^ in bin composters even though we ensured that it was exposed to thermophilic period similar to that of PrP^263K^ in previous field-scale composters. We observed up to a 3–4 log_10_ reduction in PrP^BSE^ seeding activity in lab-scale composters as opposed to only a 1–2 log_10_ reduction in bin composters. Several factors could have contributed to these differing observations. Due to the large volume of BSE brain materials needed for this project, we used two different sources of PrP^BSE^ in two composting systems. The lab-scale composting experiment used brain tissues from a cattle intra-cranially challenged with infectious BH from a Canadian BSE field case, while materials used for bin composting experiment were from a cattle orally challenged using the brain tissues from the intra-cranially challenged animal used in lab-scale composting experiment. Moreover, brain tissues used for lab-scale composting experiment were strongly positive for PrP^BSE^ with limit of detection at 30 µg by WB (Supplementary Figs. S1a and S5a), but materials used to make the homogenate in the bin composting experiment were moderate to strongly PrP^BSE^ positive which was detected at the limit of 300 µg (Figs. S1a and S7a). This suggests a higher concentration of PrP^BSE^ in the brain tissues used in lab-scale than bin composting experiment, which is also reflected by the fact that one more PMCA round is required for PrP^BSE^ amplification in bin composting experiment brain tissues to achieve a sensitivity similar to that obtain in the brain tissues used in lab-scale composting experiment. Therefore, varying concentrations of PrP^BSE^ in the infectious BH or even possibly difference in PrP^BSE^ stability related to inoculation route or host variability (i.e., PrP expression level, co-factor variation/expression, breed composition etc.) may have impacted PrP^BSE^ degradation during composting. Giles et al.^64^ reported that cattle PrP^BSE^ was 1,000-fold more resistant to inactivation by acidic SDS treatment than mouse adapted PrP^BSE^ 301V. In addition, we used two different sources of feedlot manure in two composting experiments. The fresh manure used for lab-scale composting experiment significantly had lower moisture and pH but higher TC content than the one in the bin composting experiment. Therefore, proteolytic microbial populations developed in lab-scale composters may have differed from those established in bin composters, particularly in lab-scale composters that were enriched for keratinolytic bacteria via the addition of feathers. Based on these results, temperature can be used as a proxy for microbial activity in compost, but lacks merit as direct indicator of PrP^BSE^ degradation. Therefore, further investigation is needed to characterize the biodegradation of PrP^BSE^ with consideration for other complex factors such as differences in PrP^BSE^ strains and the proteolytic microbial communities in compost.

In general, thermophilic composting (i.e., temperature ≥55°C) has a hierarchy of essential factors that facilitate the biodegradation of waste materials and inactivation of pathogens. In this study, we successfully stimulated thermophilic temperature profiles that were similar to that observed during field-scale composting^18^. However, our composting model in biocontainment did not fully represent the field scale composting systems that could be used for the disposal of SRM or cattle carcasses. In containment, ~2 tonnes of feedlot manure were used over 120 days of composting, resulting in a ~1 to 2 log_10_ PrP^BSE^ infectivity reduction. In contrast, in our field scale model^18^, ~100 tonnes of feedlot manure with 16 cattle carcasses were composted over 230 days resulting in a 4.8 log_10_ reduction in PrP^263^^K^ infectivity. As a logical extrapolation, a longer composting duration and greater volume of biomass in the field-scale composters would likely enhance PrP^BSE^ degradation. As a 4.8 log_10_ reduction of PrP^263K^ infectivity was observed in the field-scale composters^18^, it would be surprising if inactivation of PrP^BSE^ was not further enhanced in field-scale composting. In addition, Belondrade et al.^65,66^ demonstrated that commercial chemicals fully efficient on sterilization of PrP^263K^ were inefficient for the inactivation of variant PrP^CJD^, suggesting PrP^263K^ might not be a suitable model to validate the prion resistance to inactivation. Consequently, further investigation of PrP^BSE^ degradation in field-scale composting is needed.

Previous studies documented the more recalcitrant nature of PrP^BSE^ than other TSE agents. After exposure to acidic SDS, PrP^BSE^ was 10 and 10 million fold more resistant to inactivation than PrP^CJD^ and hamster PrP^Sc^, respectively, as assessed by infectivity titration in transgenic mice^64^. Langeveld et al.^67^ also reported PrP^BSE^ to be more resistant to wet heat conditions at 115°C than PrP^263^^K^ and PrP^CWD^ as measured by transgenic mouse bioassay. Our PMCA results suggested that 28 days of lab-scale composting resulted in a reduction of PrP^TSE^ seeding capacity with ~ 2 log_10_ in PrP^263K^ and ~ 3 log_10_ in PrP^CWD^ in a previous study^18^ and ~ 3 log_10_ in PrP^BSE^ in the current study. Different from chemical treatment of prion inactivation, compost is an exceedingly complex biological system, owning to changing temperatures and pH, and dynamic changes in microbial communities and the enzymes they produce during composting. Once PrP^TSE^ enter the compost environment, a wide variety of physicochemical and microbiological processes can impact PrP^TSE^ infectivity and seeding capacity. These uncontrolled factors might help to account for the variable inactivation observed in our PrP^TSE^ composting studies. While this variability calls into question the utility of our composters for complete PrP^TSE^ inactivation, it is encouraging that when our compost conditions were optimal, 28 days of composting effectively destructed PrP^BSE^ replication capacity in vitro by 3 log_10_ (i.e., at least 99.9%). Currently, the Canadian government^68^ enacted a regulation on the limited use of composting for disposal of SRM under a temporary permit. It also requires a 5-year respite from cattle access to pasture or grazing land amended with SRM compost and from direct human consumption of annual crops produced from SRM compost amended field^69^. A recent study from UK^7^ reported that the same amount of PrP^BSE^ infectivity remained in both clay and sandy soil over a 5-year period. Our studies suggests that the maximum PrP^BSE^ degradation (up to 3–4 log_10_) can achieve in the lab-scale composters with the addition of feathers. Therefore, composting of BSE infected-SRM prior to subsequent land application could be an effective approach to reduce the risk of high titer PrP^BSE^ persisting in the environment.

## Conclusions

In this study, we successfully quantified PrP^BSE^ degradation using PMCA and bioassay in two-scale composting systems. After 28 days, ~ 3 log_10_ reduction of PrP^BSE^ seeding activity was observed in lab-scale composters. Addition of chicken feathers to the compost enhanced PrP^BSE^ degradation, likely as a result of enrichment for keratinolytic bacteria. After 106–120 days, both BSE associated seeding activity and infectivity were reduced by 1–2 log_10_ in the centre, but only by 1 log_10_ at the bottom of bin composters. This suggests that placement of SRM in the centre of compost piles would be more amendable for the biodegradation of PrP^BSE^. Current CFIA policy on SRM destruction methods require at least 5 log_10_ reduction of PrP^BSE^ to approve composting for disposal of BSE positive SRM. Our field-scale composting study^18^ demonstrated that 230 days of composting resulted in a 4.8 log_10_ inactivation in hamster PrP^263^^K^ infectivity. However, PrP^263K^ might be not a suitable surrogate model to validate the PrP^BSE^ resistance in compost. The outcomes generated from this study did not meet this criteria, but do lay the foundational work needed to further optimize the degradation of PrP^BSE^ in compost. Spatial variability in microbial activity within static compost piles makes it unlikely that the procedure will ever achieve the 5 log_10_ reduction in PrP^BSE^ required for full regulatory approval as a disposal method of SRM.

## Supplementary Information

Supplementary Information.

## Data Availability

The datasets generated during and/or analyzed during the current study are available from the corresponding author on reasonable request.
