# Supplementary material for: Biodegradation of bovine spongiform encephalopathy prions in compost
Source: Sci Rep. 2022 Dec 23;12:22233. doi: 10.1038/s41598-022-26201-2 (PMC9789035; doi:10.1038/s41598-022-26201-2)
Supplement: Supplementary file 1 — Supplementary Information. [file 41598_2022_26201_MOESM1_ESM.docx]

**Supplementary information**

Biodegradation of bovine spongiform encephalopathy prions in compost

Shanwei Xu, Sujeema Abeysekara, Sandor Dudas, Stefanie Czub, Antanas Staskevicius, Gordon Mitchell, Kingsley K. Amoako & Tim A. McAllister

**Table S1.** Changes of compost pH and moisture content during lab-scale and bin composting.

|  |  | Lab-scale compost^a^ | | |  | | | | | | | Bin compost | | | | | |  |  |  |  |  |
| --- | --- | --- | --- | --- | --- | --- | --- | --- | --- | --- | --- | --- | --- | --- | --- | --- | --- | --- | --- | --- | --- | --- |
| Compost parameters |  | Control  compost |  | Feather  compost |  | | | 2017 Experiment | | | | | | 2018 Experiment | | | |  |  |  |  |  |
|  |  |  |  |  |  | | |  |  |  |  |  |  |  |  |  |  |  |  |  |  |  |
| Moisture (%) |  |  | | | |  |  | |  | |  | | | | |  |  | |  | |  |  |
| After 1^st^ mixing |  | 64.3 ± 1.6^b^ |  | 63.2 ± 2.1 |  | | | | | 69.6 ± 1.1 | | | | | 62.2 ± 3.5 | | | |  |  |  |  |
| After 2^nd^ mixing |  | NA^c^ |  | NA |  | | | | | 59.7 ± 3.3 | | | | | 59.4 ± 2.9 | | | |  |  |  |  |
| After experiment |  | 54.0 ± 1.0 |  | 49.5 ± 0.8 |  | | | | | 54.5 ± 2.5 | | | | | 52.4 ± 2.1 | | | |  |  |  |  |
| pH |  |  | | | |  |  | |  | |  | |  | | | | | |  |  |  |  |
| After 1^st^ mixing |  | 9.2 ± 0.5 |  | 9.5 ± 0.1 |  | | | | | 9.2 ± 0.1 | | | | | 9.1 ± 0.0 | | | |  |  |  |  |
| After 2^nd^ mixing |  | NA |  | NA |  | | | | | 8.5 ± 0.2b^d^ | | | | | 8.8 ± 0.3a | | | |  |  |  |  |
| After experiment |  | 9.6 ± 0.1 |  | 9.7 ± 0.1 |  | | | | | 8.4 ± 0.3 | | | | | 8.6 ± 0.1 | | | |  |  |  |  |

^a^Data was cited from Xu et al.^18^.

^b^ Mean ± SEM (n = 3 for lab-scale compost; n = 2 for bin compost).

^c^NA: Not applicable.

^d^Within a row, values followed by different lower case letters differ (*P* < 0.05) between two experimental years at the same sampling day in bin composting experiment.

**Table S2.** Titration assays of the stainless steel wires coated with the 10-fold dilutions of 10% original BSE brain homogenates used in 2017 and 2018 bin composting experiments.

| Dilution factor | Tgbov XV mice  (2017 Experiment ) | | | | |  | |  | Tgbov XV mice  (2018 Experiment ) | | | | | | | | |
| --- | --- | --- | --- | --- | --- | --- | --- | --- | --- | --- | --- | --- | --- | --- | --- | --- | --- |
|  | Survival period in dpi^a^  (mean ± SEM) | | | Attack rate^b^ | | |  | | | Survival period in dpi  (mean ± SEM) | | | |  | | Attack rate |  |
|  |  |  |  | |  | |  | | | |  |  |  | |  | | |
| 10^-1^ | 425 ± 14 |  |  | | 7/7 | |  | | | | 385 ± 8 |  |  | | 8/8 | | |
| 10^-2^ | 445 ± 9 |  |  | | 7/7 | |  | | | | 458 ± 10 |  |  | | 8/8 | | |
| 10^-3^ | 440 ± 8 |  |  | | 8/8 | |  | | | | 539 ± 25 |  |  | | 6/8 | | |
| 10^-4^ | 453 ± 22 |  |  | | 8/8 | |  | | | |  |  |  | | 0/6 | | |
| 10^-5^ | 462 ± 15 |  |  | | 6/6 | |  | | | | 525 |  |  | | 1/6 | | |
| 10^-6^ | 544 ± 26 |  |  | | 6/7 | |  | | | |  |  |  | | 0/7 | | |
| 10^-7^ |  |  |  | | 0/6 | |  | | | |  |  |  | | 0/6 | | |
| 10^-8^ |  |  |  | | 0/6 | |  | | | |  |  |  | | 0/6 | | |
| 10^-9^ |  |  |  | | 0/7 | |  | | | |  |  |  | | 0/6 | | |
| Positive control | 432 ± 9 |  |  | | 7/7 | |  | | | | 394 ± 4 |  |  | | 7/7 | | |
| Negative control |  |  |  | | 0/6 | |  | | | |  |  |  | | 0/7 | | |

^a^Survival period: Number of days post inoculation (dpi) when mice died or were euthanized with clinical signs of neurological disease. Mice euthanized due to concurrent illness or that tested TSE-negative were not included in calculations. Mean values are not presented if all mice in a group were TSE-negative.

^b^Attack rate: Number of TSE-positive mice / Number of mice challenged.

**
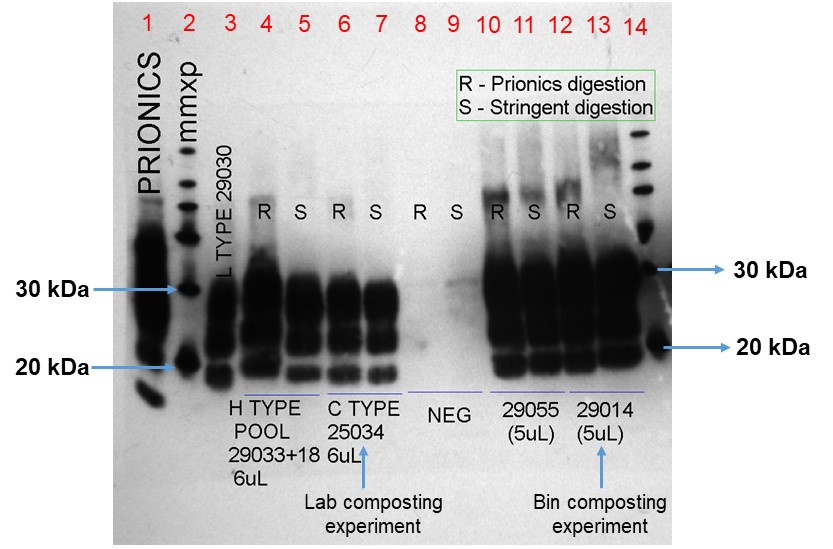
**

(a)

(b)

(c)


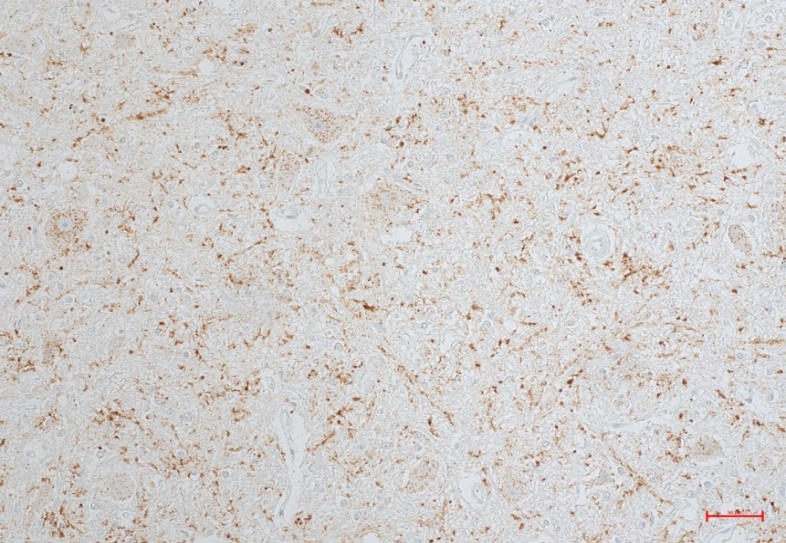


50 µm


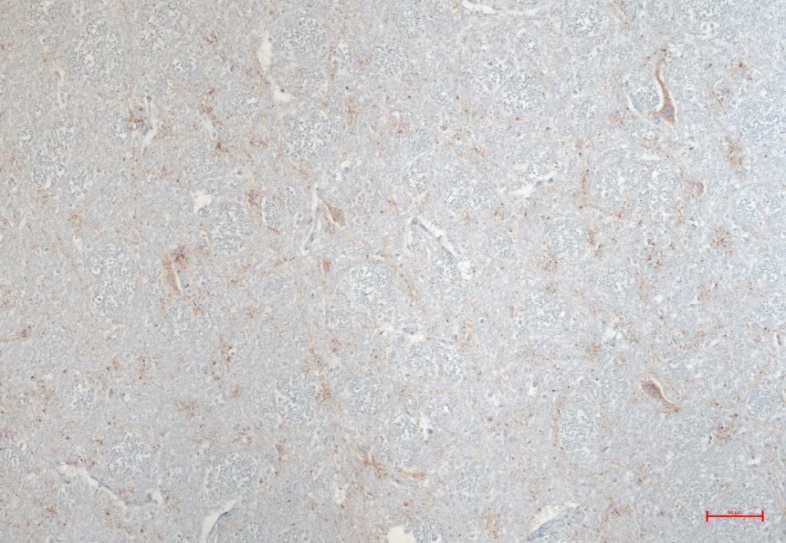


50 µm

**Figure S1.** (a) Western blot immunodetection of proteinase K (PK)-resistant PrP^BSE^ using core antibody 6H4 in the brainstem samples of BSE-infected cattle used in lab-scale (Lanes 6 and 7 containing 600 µg of original brain materials) and bin (Lanes 12 and 13 containing 500 µg of original brain materials) composting experiments. Both brainstem samples were confirmed to have C-type BSE glycoform profile as compared to L-type (Lane 3) and H-type (Lanes 4 and 5) BSE positive brain materials. Lane 1: Western blot kit control, normal bovine brain homogenate. R: Regular PK digestion using Prionics-Check Western digestion buffer and PK reagent at 48⁰C for 40 min from Prionics-Check Western blot kit; S: Stringent PK digestion using stringent digestion buffer (1M Tris-HCl, pH 8.0) and 833 µg mL^-1^ PK at 48⁰C for 40 min. Molecular weight markers at 30 kDa (Upper ladder) and 20 kDa (Lower ladder) are indicated. (b) and (c): Immunohistochemistry images at 20X magnification using antibody F99 for detection of PrP^BSE^ on the deep cerebellar nuclei of bovine brain tissues used in lab-scale and bin composting experiment, respectively. Both figures show strong staining of prion aggregates indicative of classical BSE.

(a)

(b)

(c)


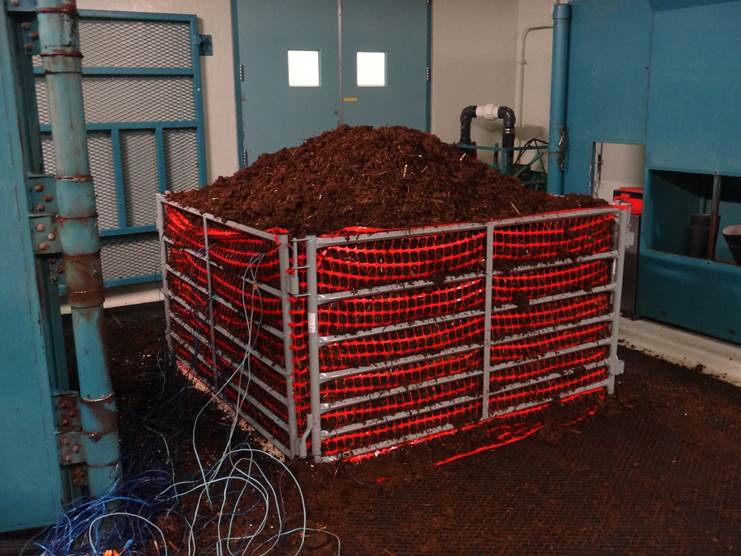


**Figure S2.** (a) Schematics of layout (Top view) of a biocontainment level 3 room for construction of compost bins at CFIA, Lethbridge, AB. (b) Duplicate bins (1.8 m length × 1.8 m width × 1.2 m height) were constructed using eight metal fence panels surrounded by snow fence. When compost temperature declined below 50°C, the compost was mixed and moistened with carbon added as needed and the mixture was transferred to a second bin for the next heating cycle. A total of three heating cycles were generated over the experiment. Wood shavings (6 cm) were placed on the floor prior to introduction of manure into the bin. An average initial wet weight of ~2,200 kg fresh manure was then placed on top of the wood shavings, forming a dome 1.5-m high compost pile. (c) Schematic of the nine locations of temperature measurement at the bottom (36 cm), centre (72 cm) and top (108 cm) layer within bin composters. Mesh bags containing manure spheres or stainless steel wires inoculated with negative and positive PrP^BSE^ were placed at the centre layer (Location 5) of bin composters in 2017 and at both centre and bottom layers (Location 5) of bin composters in 2018. Units in cm.


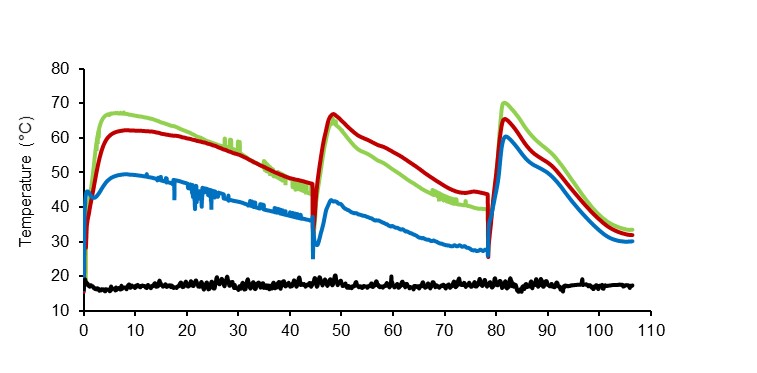

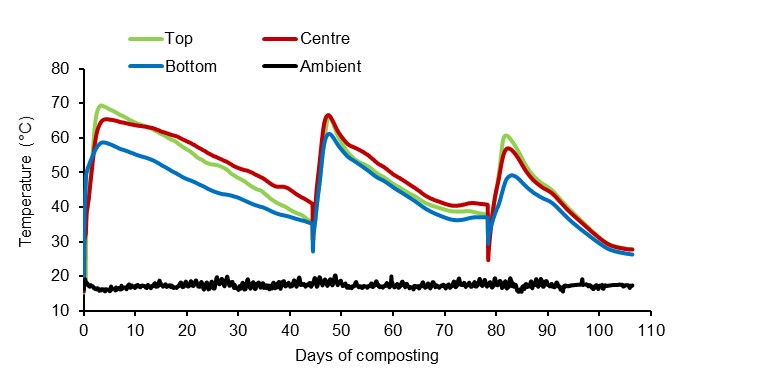

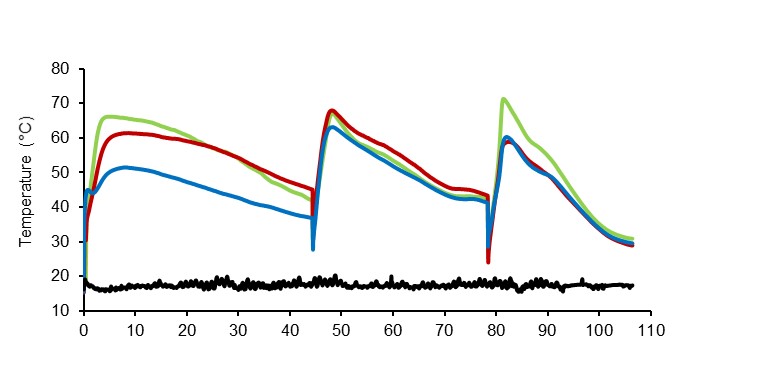


(a) Location 1 – 2017 Experiment

(b) Location 2 - 2017 Experiment


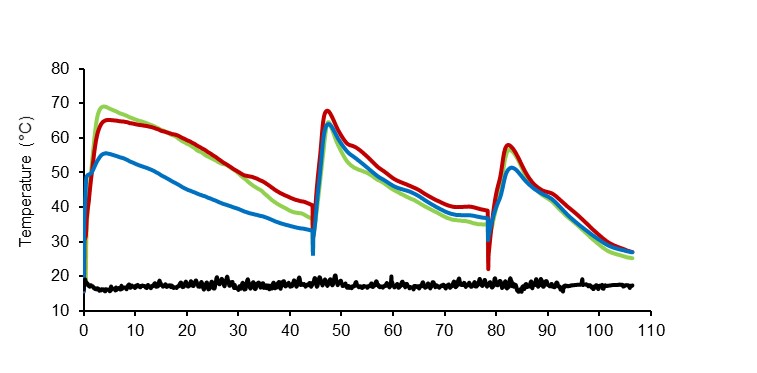


(c) Location 3 - 2017 Experiment

(d) Location 4 - 2017 Experiment


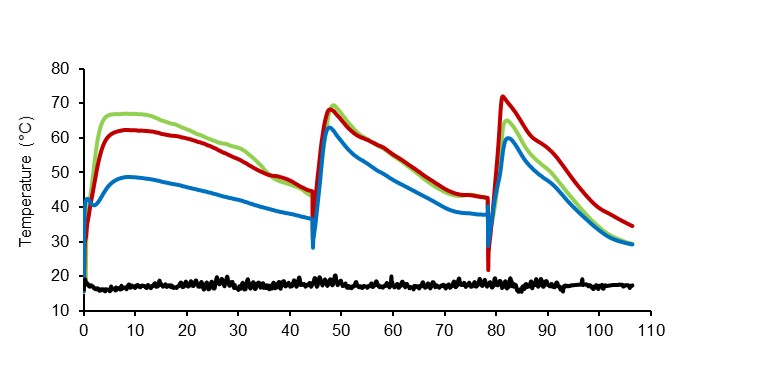

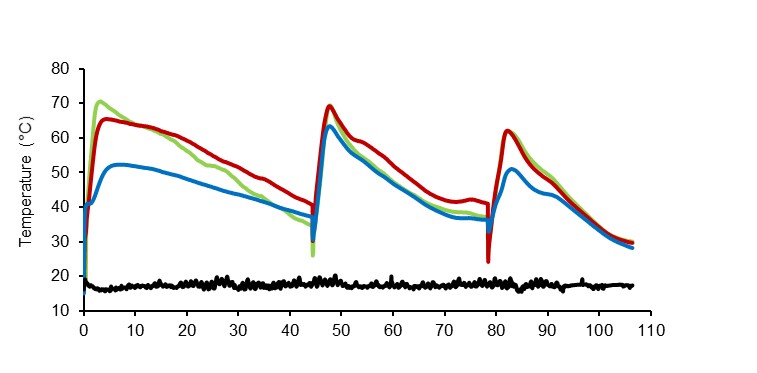

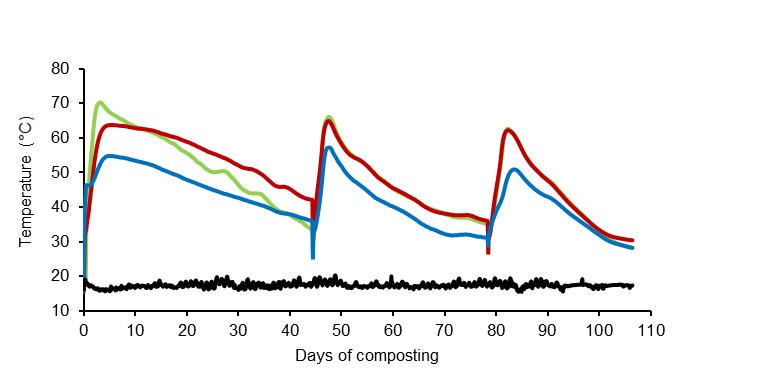


(e) Location 6 - 2017 Experiment

(f) Location 7 - 2017 Experiment

(g) Location 8 - 2017 Experiment

(h) Location 9 - 2017 Experiment

**Figure S3.** Temperature profiles from the edge locations (i.e., Locations 1-4 and 6-9 in Figure S2c) at top (108 cm), centre (72 cm) and bottom (36 cm) layers of bin composters over 106 days in 2017. Compost was mixed two times on days 44 and 78 for bin composters. The duration days of temperatures ≥55°C at all three layers of bin composters were shown in each figure. Arrows indicate the date compost was mixed.


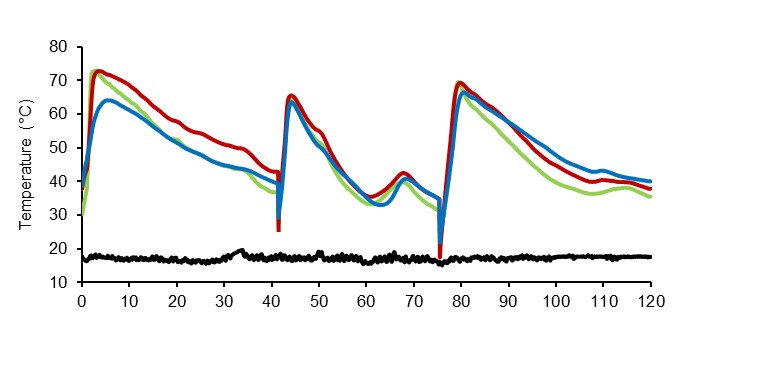

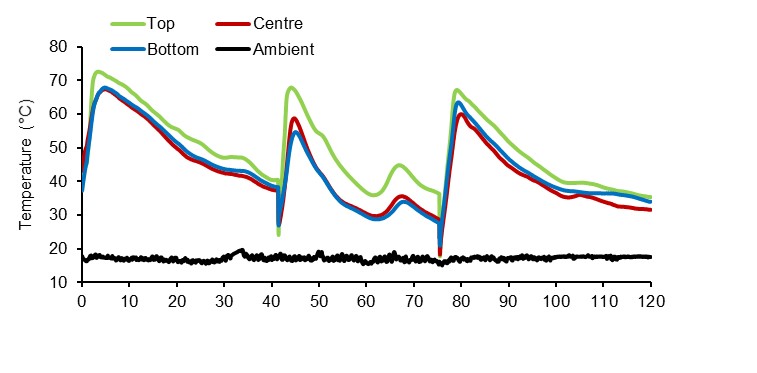

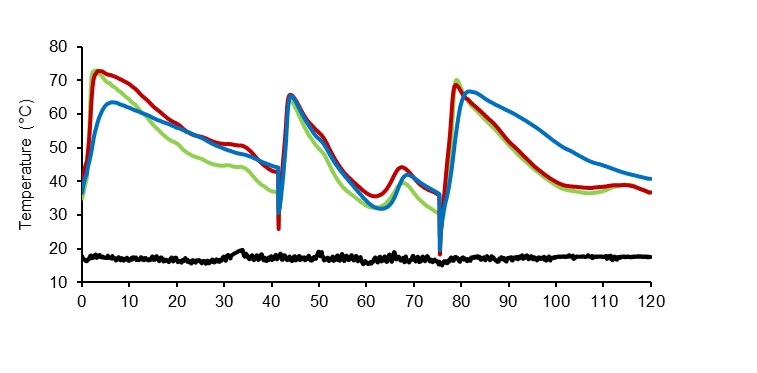


(a) Location 1 – 2018 Experiment

(b) Location 2 – 2018 Experiment

(c) Location 3 – 2018 Experiment

(d) Location 4 – 2018 Experiment


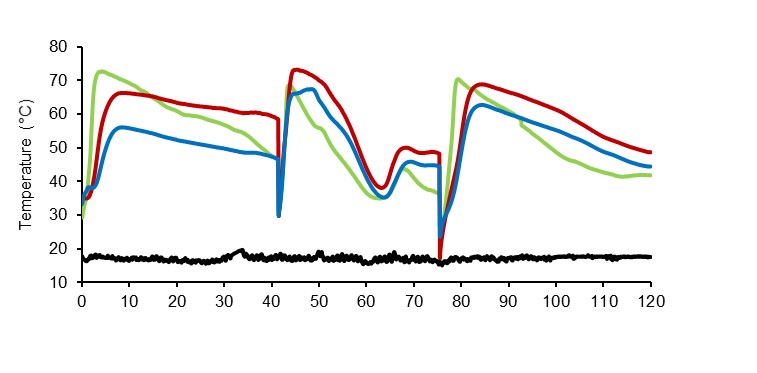

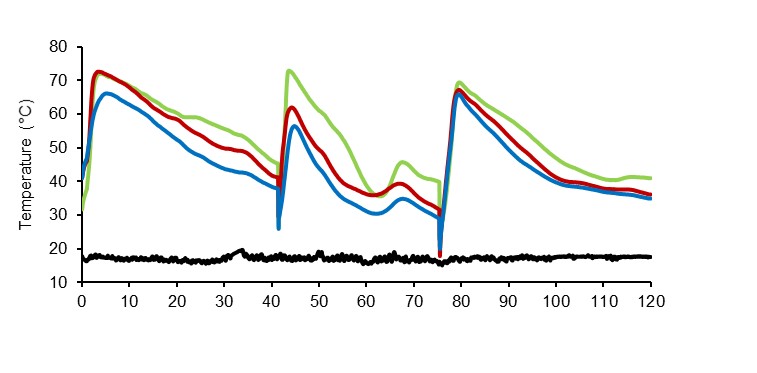

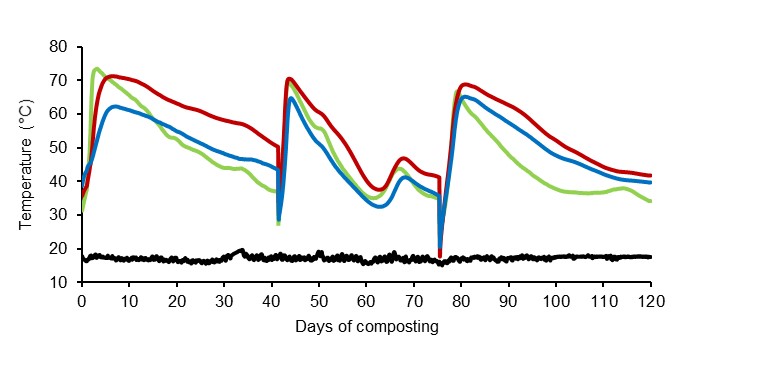


(e) Location 6 – 2018 Experiment

(f) Location 7 – 2018 Experiment

(h) Location 9 – 2018 Experiment

(g) Location 8 – 2018 Experiment

**Figure S4.** Temperature profiles from the edge locations (i.e., Locations 1-4 and 6-9 in Figure S2c) at top (108 cm), centre (72 cm) and bottom (36 cm) layers of bin composters over 120 days in 2018. Compost was mixed two times on days 41 and 75 for bin composters. The duration days of temperatures ≥55°C at all three layers of bin composters were shown in each figure. Arrows indicate the date compost was mixed.

**
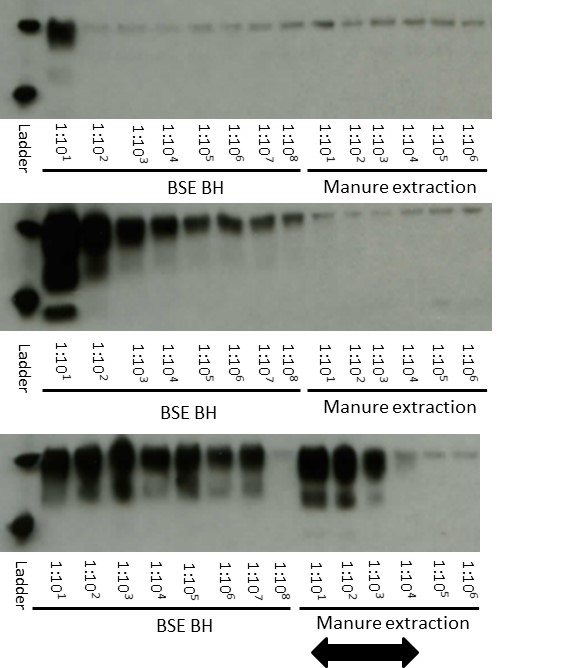
**

(a)

(b)

(c)

**Figure S5.** Representative Western blots of PrP^BSE^ extracted from fresh manure spheres inoculated with decreasing concentrations of 10% BSE brain homogenate (BH) before (a) and after the first (b) and second (c) round of protein misfolding cyclic amplification (PMCA). The left portion of each panel shows10 fold dilutions of 10% BSE BH containing 30 µg (Lane: 1:10), 3 µg (Lane: 1:10^2^), 0.3 µg (Lane: 1:10^3^), 0.03 µg (Lane: 1:10^4^), 3 ng (Lane: 1:10^5^), 0.3 ng (Lane: 1:10^6^), 0.03 ng (Lane: 1:10^7^) and 0.003 ng (Lane: 1:10^8^) of original BSE positive materials, verifying PMCA amplification. Brain tissues were detected at the limit of 30 µg before PMCA. Arrows indicate the dilution range where PrP^BSE^ signals were detected in manure extraction samples after the second round of PMCA. Molecular weight markers at 30 kDa (Upper ladder) and 20 kDa (Lower ladder) are indicated. The blot for each figure was cropped from the same gel. Original blots are presented in Supplementary Fig. S12.


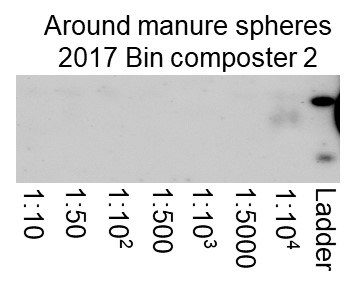

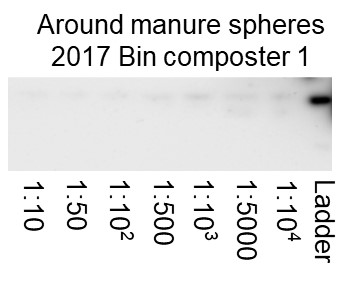


(a)


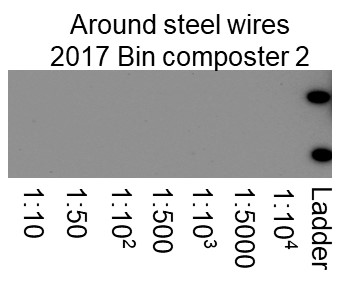

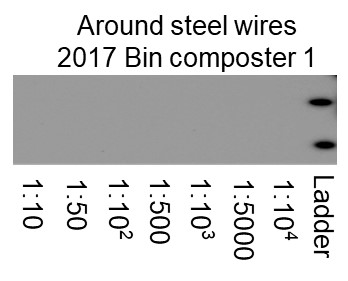


(b)

(c)


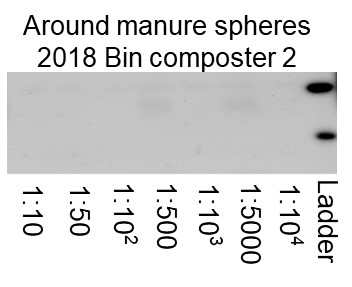

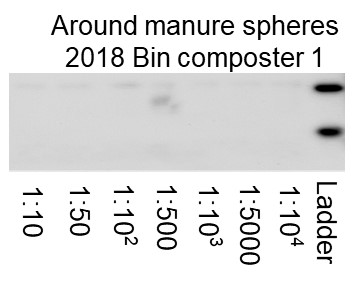


(d)


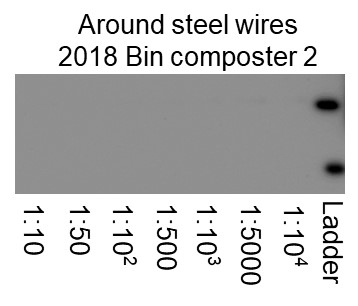

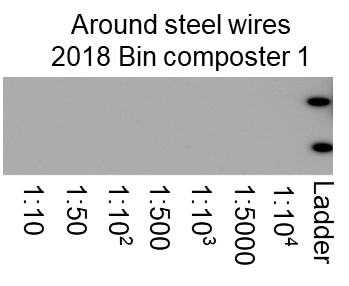


**Figure S6.** Representative Western blots of PrP^BSE^ extracted from the composite compost samples adjacent to nylon bags containing manure spheres or stainless steel wires in 2017 (a and b) and 2018 (c and d) bin composting experiments after three rounds of protein misfolding cyclic amplification (PMCA). All compost samples were collected from the centre layer of the bin composters at the end of the experiment. Molecular weight markers at 30 kDa (Upper ladder) and 20 kDa (Lower ladder) are indicated. The blots for each figure were cropped from the same gel. Original blots are presented in Supplementary Fig. S13.


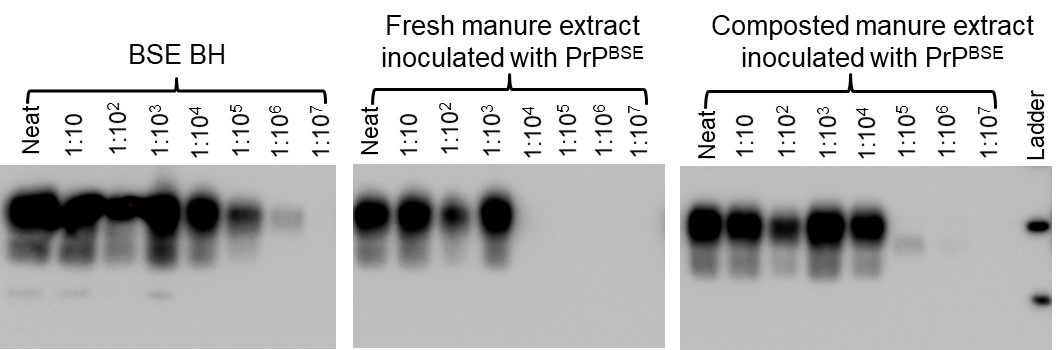

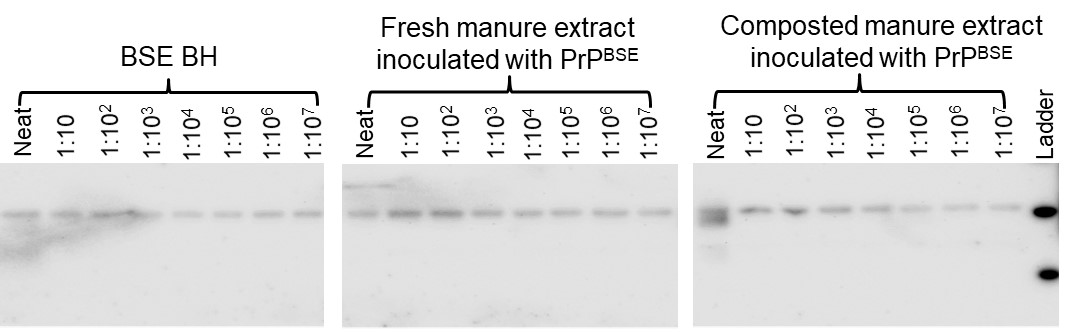


(a)

(b)

**Figure S7.** Representative Western blots of PrP^BSE^ extracted from fresh and composted manure spheres inoculated with decreasing concentrations of 10% BSE brain homogenate (BH) before (a) and after (b) three rounds of protein misfolding cyclic amplification (PMCA). The left panel shows 10 fold dilutions of 10% BSE BH containing 300 µg (Lane: Neat), 30 µg (Lane: 1:10), 3 µg (Lane: 1:10^2^), 0.3 µg (Lane: 1:10^3^), 0.03 µg (Lane: 1:10^4^), 3 ng (Lane: 1:10^5^), 0.3 ng (Lane: 1:10^6^) and 0.03 ng (Lane: 1:10^7^) of original BSE positive materials, verifying PMCA amplification. Brain tissues were detected at the limit of 300 µg before PMCA. Molecular weight markers at 30 kDa (Upper ladder) and 20 kDa (Lower ladder) are indicated. The grouped blots were cropped from different parts of the same gel. Original blot is presented in Supplementary Fig. S14.

(a)

(b)


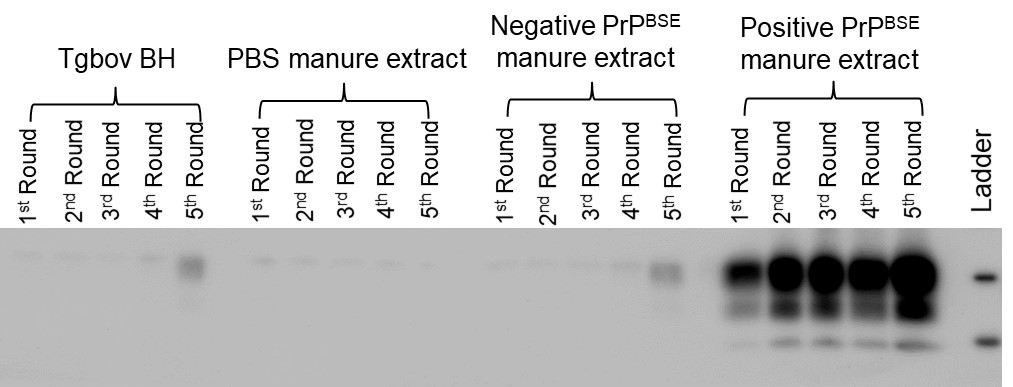

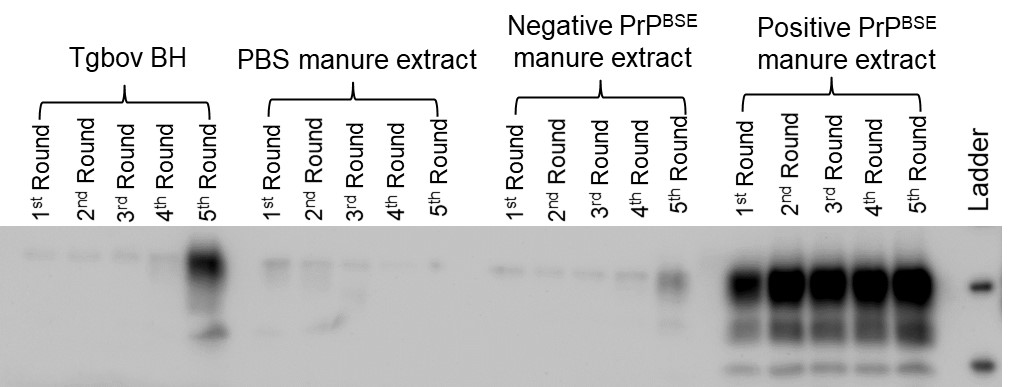


**Figure S8.** Representative Western blots of duplicate samples (a and b) from 10% Tgbov XV brain homogenate (BH) and fresh manure extraction inoculated with PBS buffer, 10% BSE negative BH, and 10% BSE positive BH after five rounds of protein misfolding cyclic amplification (PMCA). Molecular weight markers at 30 kDa (Upper ladder) and 20 kDa (Lower ladder) are indicated. The blot for each figure was cropped from the same gel. Original blots are presented in Supplementary Fig. S15.


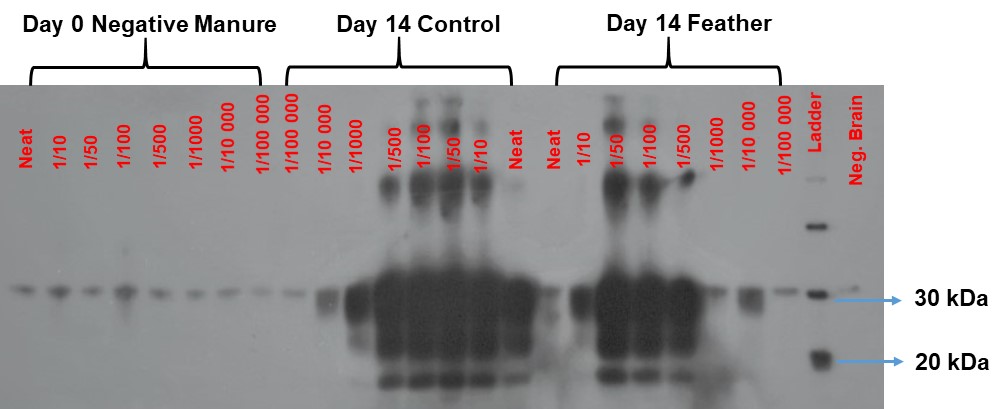


(a)


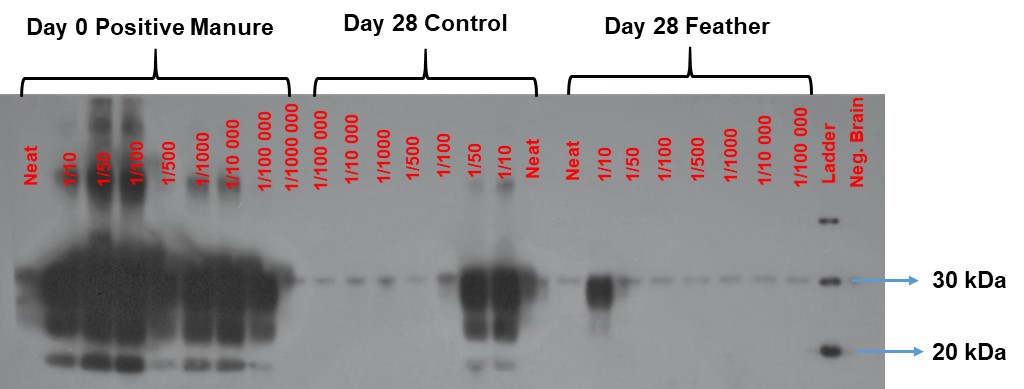


(b)

**Figure S9.** Original Western blots of PrP^BSE^ extracted from manure spheres collected at (a) days 0 and 14 and (b) days 0 and 28 from lab-scale composters after two rounds of protein misfolding cyclic amplification (PMCA). Control: control compost; Feather: feather compost. Molecular weight markers at 30 kDa (Upper ladder) and 20 kDa (Lower ladder) are indicated. Cropped blots are presented in Fig. 2.

**
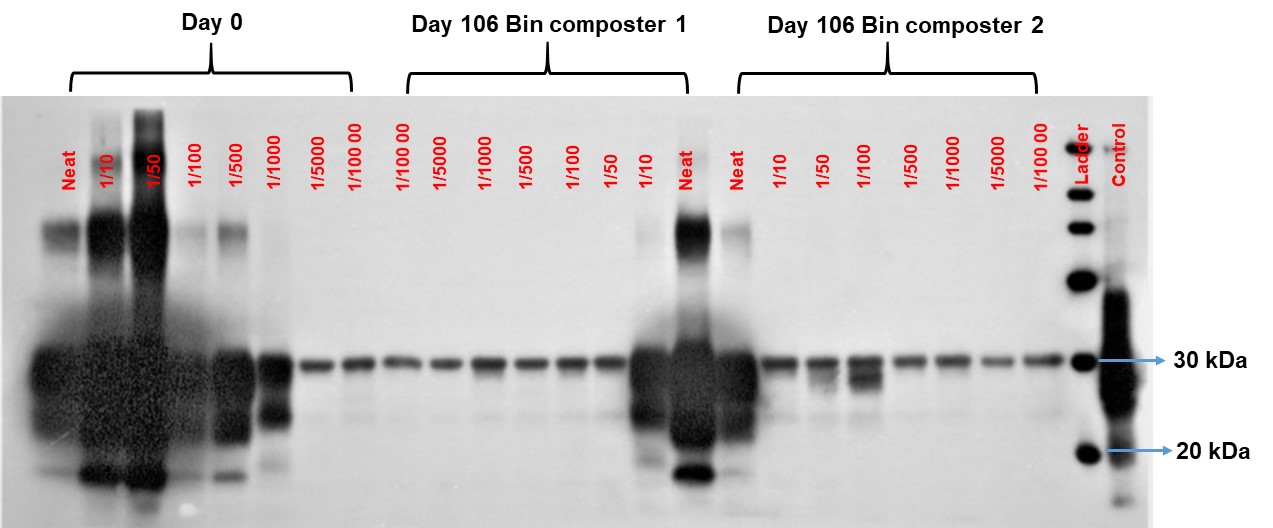
**
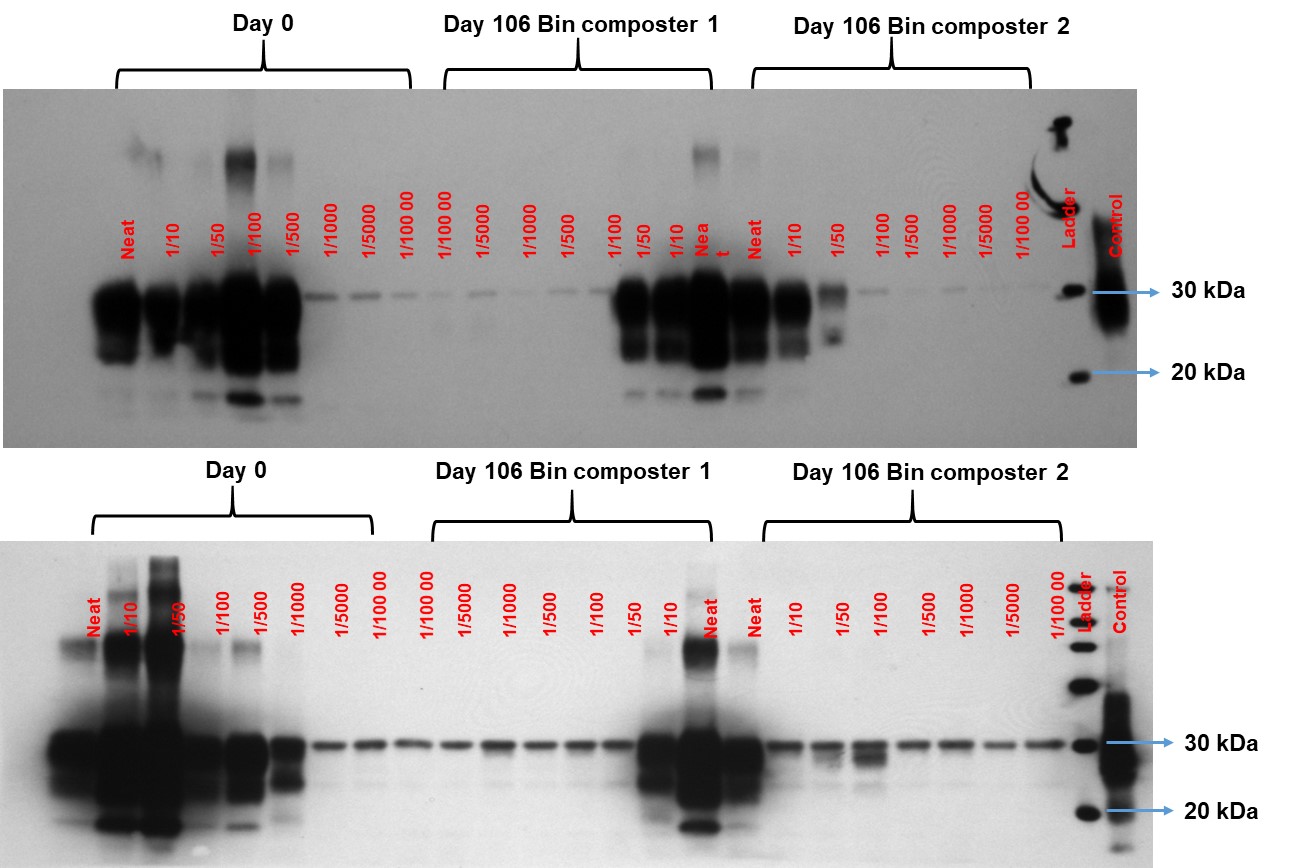


(b)

(a)

**Figure S10.** Original Western blots of PrP^BSE^ extracted from duplicate manure spheres (a) and (b) collected at days 0 and 106 from the centre layer in 2017 bin composter 1 and 2 after three rounds of protein misfolding cyclic amplification (PMCA). Molecular weight markers at 30 kDa (Upper ladder) and 20 kDa (Lower ladder) are indicated. Control: Western blot kit control, normal bovine brain homogenate. Cropped blots are presented in Fig. 3.


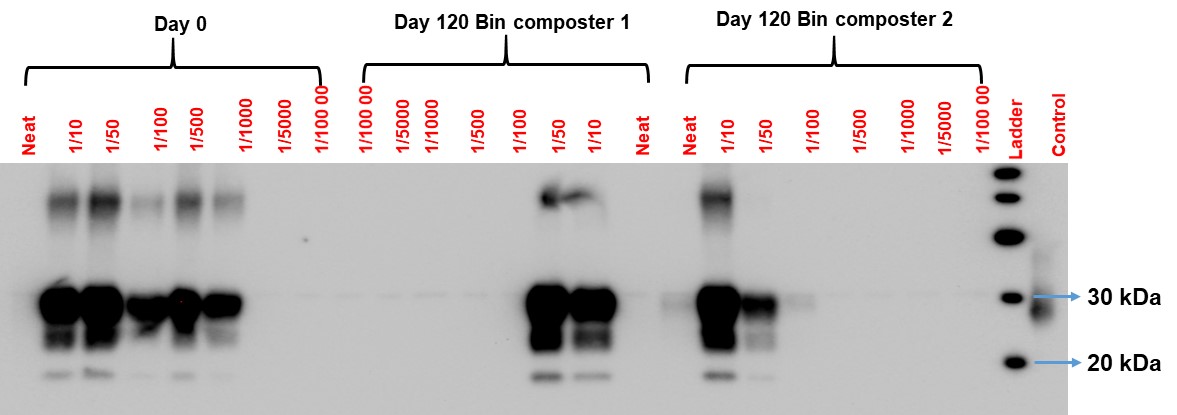


(b) Centre layer - Replicate 2

(a) Centre layer - Replicate 1


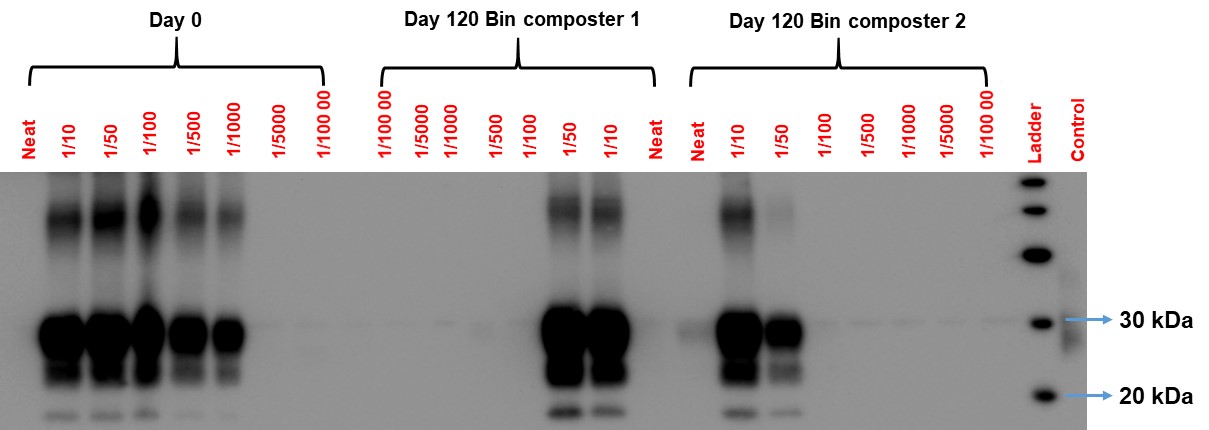


(c) Bottom layer - Replicate 1


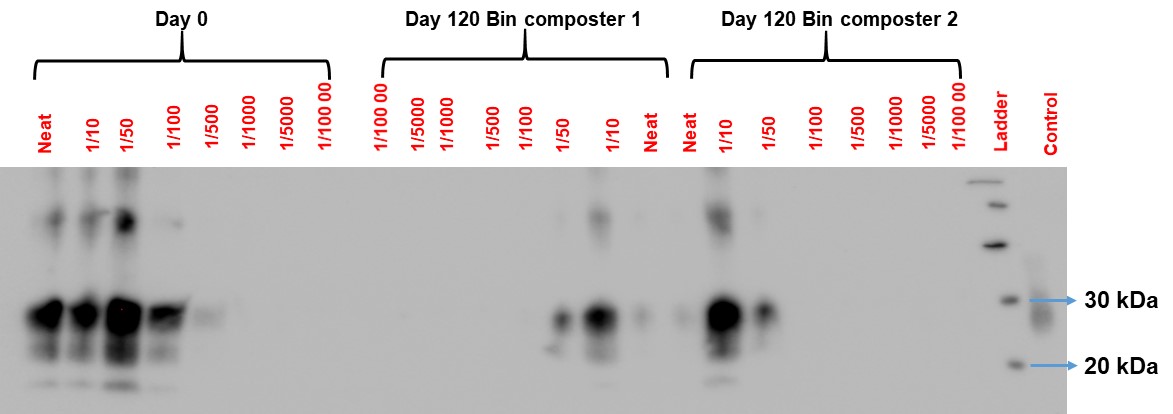


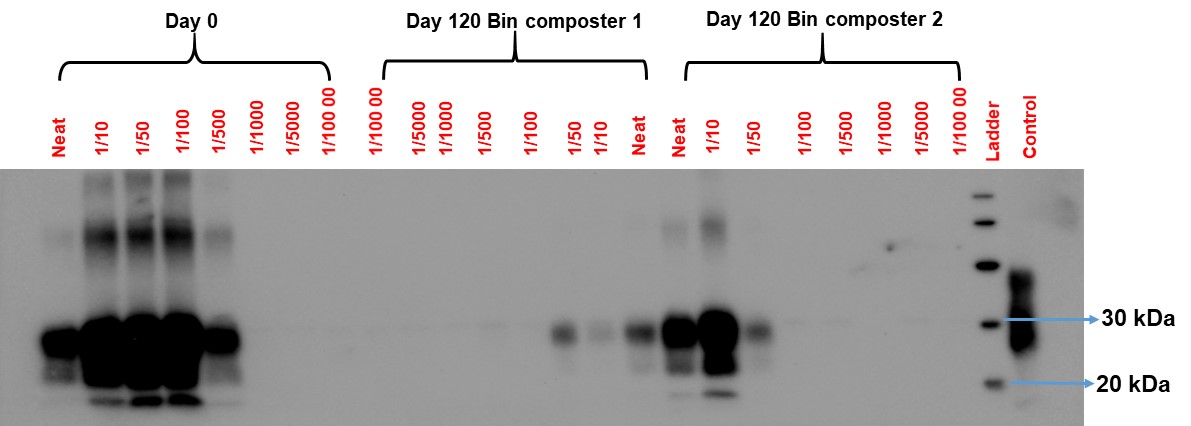


(d) Bottom layer - Replicate 2

**Figure S11.** Original Western blots of PrP^BSE^ extracted from duplicate manure spheres collected at days 0 and 120 from the centre (a and b) and bottom (c and d) layers in 2018 bin composters after three rounds of protein misfolding cyclic amplification (PMCA). Molecular weight markers at 30 kDa (Upper ladder) and 20 kDa (Lower ladder) are indicated. Control: Western blot kit control, normal bovine brain homogenate. Cropped blots are presented in Fig. 4.


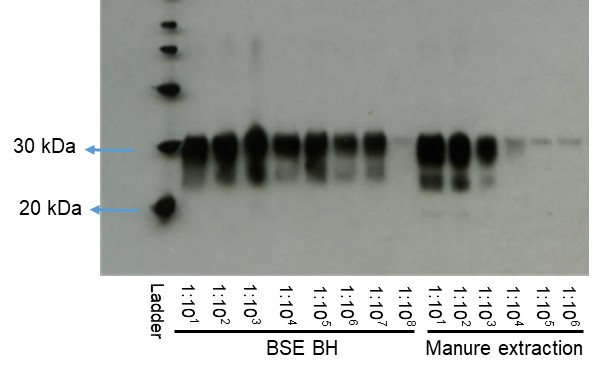

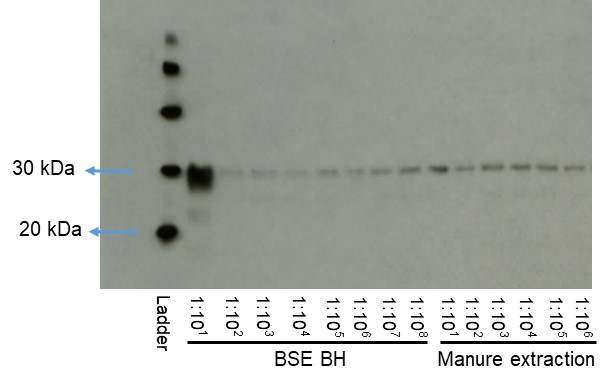

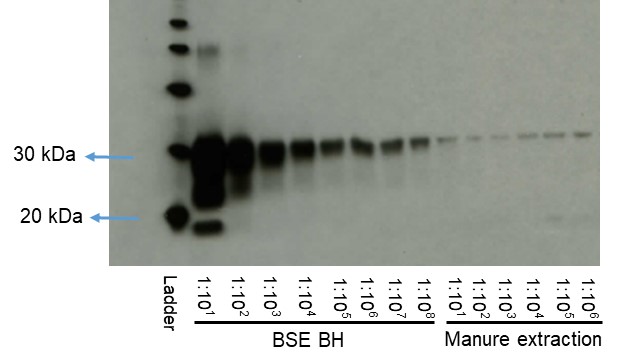


(a)

(b)

(c)

**Figure S12.** Original Western blots of PrP^BSE^ extracted from fresh manure spheres inoculated with decreasing concentrations of 10% BSE brain homogenate (BH) before (a) and after the first (b) and second (c) round of protein misfolding cyclic amplification (PMCA). The left portion of each panel shows10 fold dilutions of 10% BSE BH containing 30 µg (Lane: 1:10), 3 µg (Lane: 1:10^2^), 0.3 µg (Lane: 1:10^3^), 0.03 µg (Lane: 1:10^4^), 3 ng (Lane: 1:10^5^), 0.3 ng (Lane: 1:10^6^), 0.03 ng (Lane: 1:10^7^) and 0.003 ng (Lane: 1:10^8^) of original BSE positive materials, verifying PMCA amplification. Molecular weight markers at 30 kDa (Upper ladder) and 20 kDa (Lower ladder) are indicated. Cropped blots are presented in Supplementary Fig. S5.

(c)

(b)

(a)


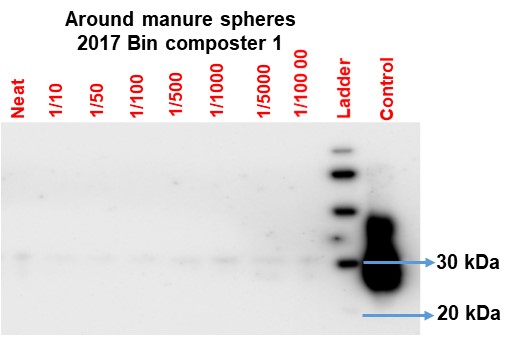

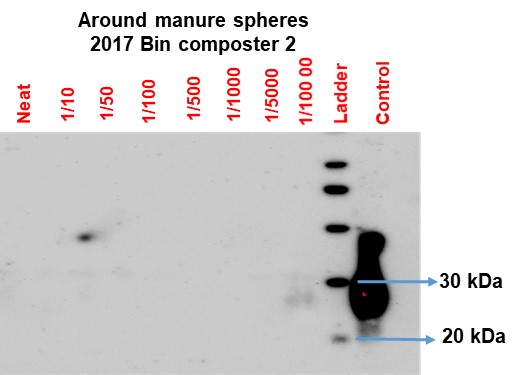

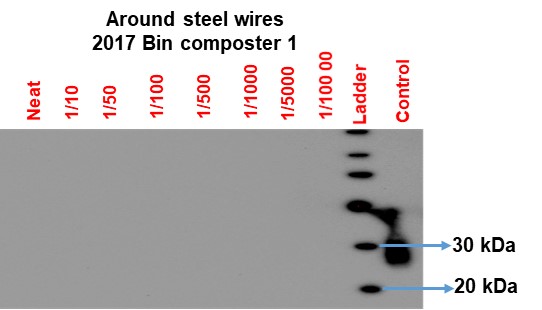

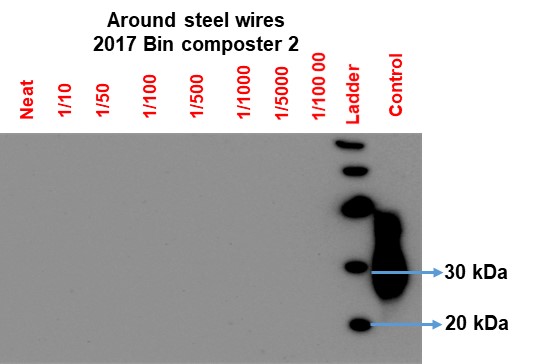

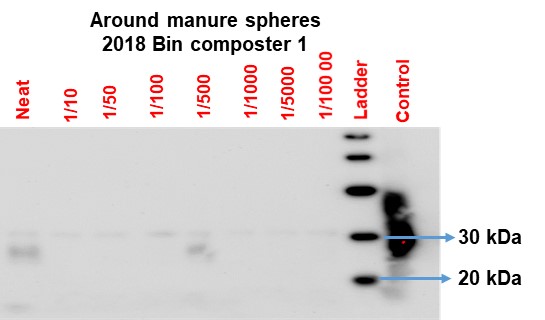

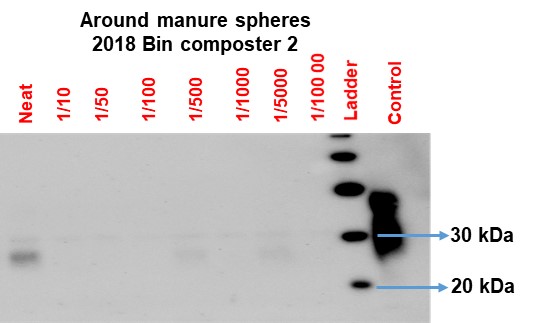


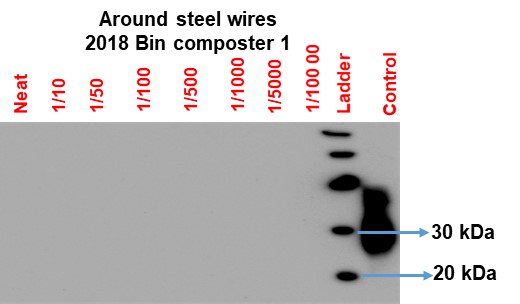

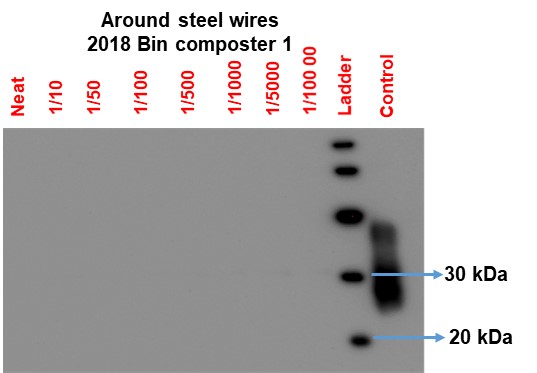


(d)

**Figure S13.** Original Western blots of PrP^BSE^ extracted from the composite compost samples adjacent to nylon bags containing manure spheres or stainless steel wires in 2017 (a and b) and 2018 (c and d) bin composting experiments after three rounds of protein misfolding cyclic amplification (PMCA). All compost samples were collected from the centre layer of the bin composters at the end of the experiment. Molecular weight markers at 30 kDa (Upper ladder) and 20 kDa (Lower ladder) are indicated. Control: Western blot kit control, normal bovine brain homogenate. Cropped blots are presented in Supplementary Fig. S6.


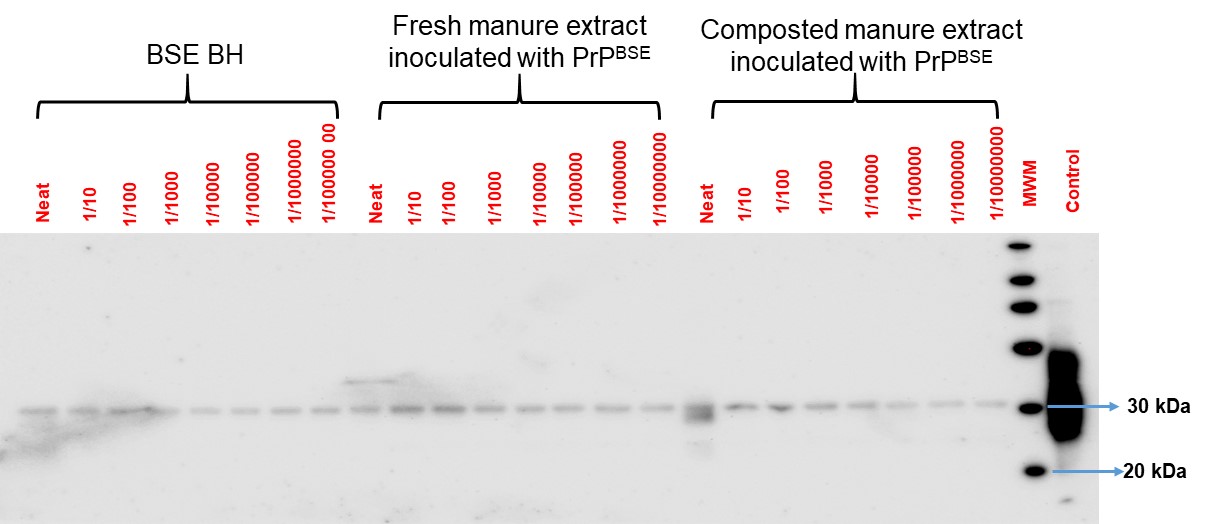

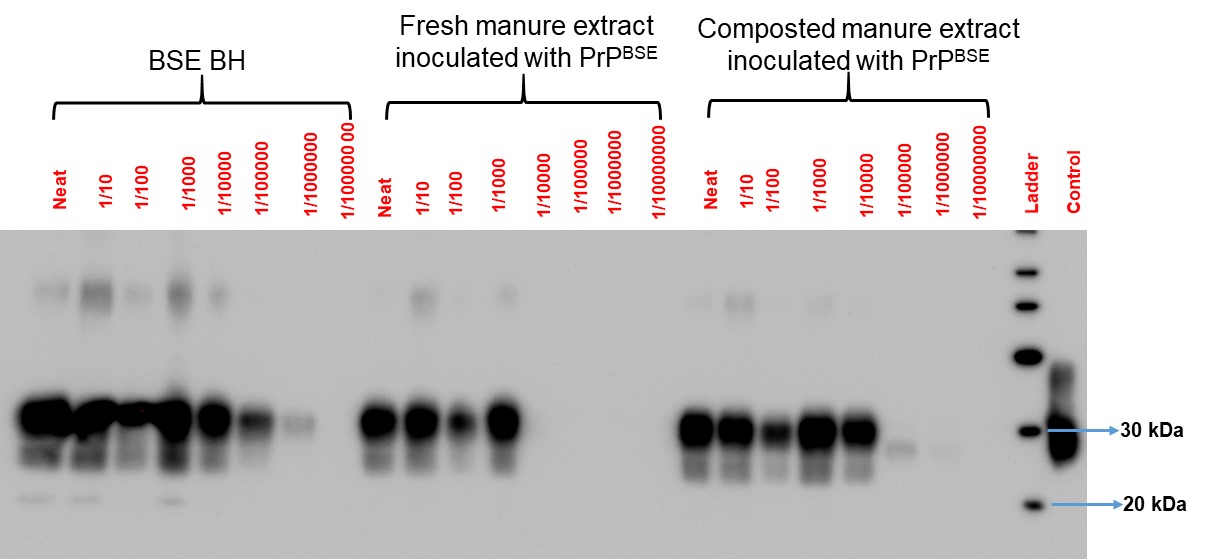


(b)

(a)

**Figure S14.** Original Western blots of PrP^BSE^ extracted from fresh and composted manure spheres inoculated with decreasing concentrations of 10% BSE brain homogenate (BH) before (a) and after (b) three rounds of protein misfolding cyclic amplification (PMCA). The left panel shows 10 fold dilutions of 10% BSE BH containing 300 µg (Lane: Neat), 30 µg (Lane: 1:10), 3 µg (Lane: 1:10^2^), 0.3 µg (Lane: 1:10^3^), 0.03 µg (Lane: 1:10^4^), 3 ng (Lane: 1:10^5^), 0.3 ng (Lane: 1:10^6^) and 0.03 ng (Lane: 1:10^7^) of original BSE positive materials, verifying PMCA amplification. Molecular weight markers at 30 kDa (Upper ladder) and 20 kDa (Lower ladder) are indicated. Control: Western blot kit control, normal bovine BH. Cropped blots are presented in Supplementary Fig. S7.


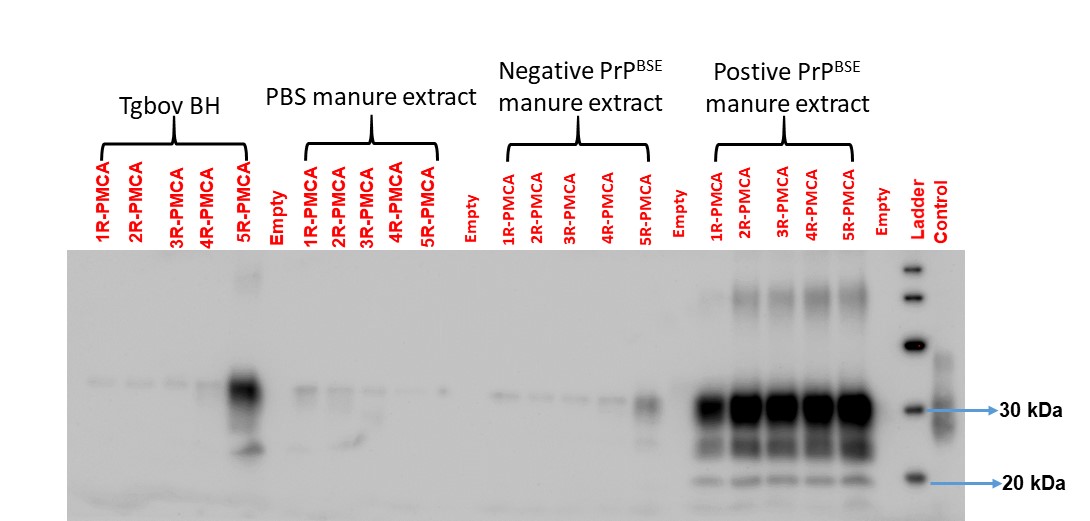

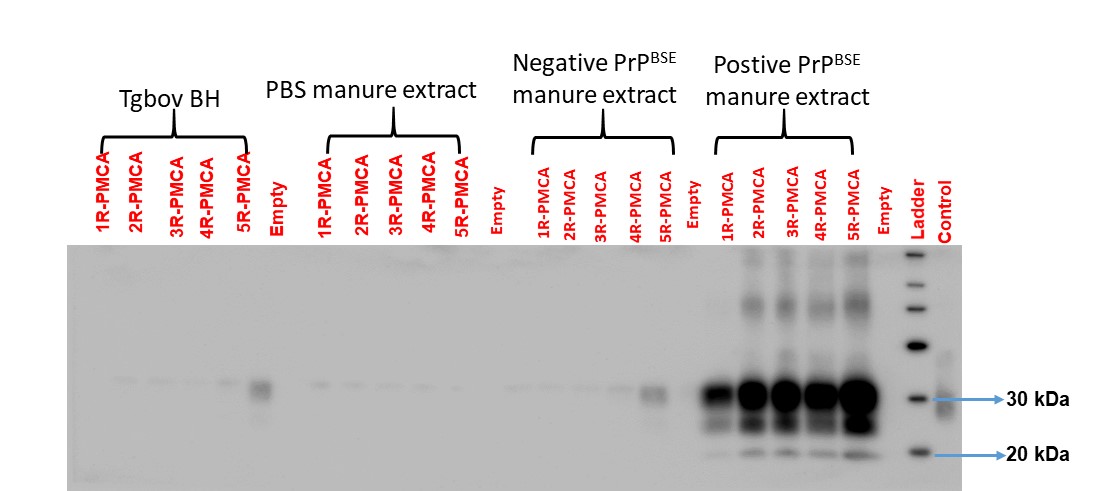


(b)

(a)

**Figure S15.** Original Western blots of duplicate samples (a and b) from 10% Tgbov XV brain homogenate (BH) and fresh manure extraction inoculated with PBS buffer, 10% BSE negative BH, and 10% BSE positive BH after five rounds of protein misfolding cyclic amplification (PMCA). Molecular weight markers at 30 kDa (Upper ladder) and 20 kDa (Lower ladder) are indicated. Control: Western blot kit control, normal bovine BH. Cropped blots are presented in Supplementary Fig. S8.
